# Mechanism of Dinitrogen Photoactivation by P_2_P^Ph^Fe Complexes: Thermodynamic and Kinetic Computational Studies

**DOI:** 10.1021/acs.inorgchem.4c04006

**Published:** 2024-10-23

**Authors:** Camilo Prada, Eugenia Dzib, Francisco Núñez-Zarur, Pedro Salvador, Gabriel Merino, Carmen J. Calzado, Jhon Zapata-Rivera

**Affiliations:** a Departamento de Química, Universidad de los Andes, Cra 1 No. 18A − 12, Bogotá 111711, Colombia; b Departamento de Física Aplicada, Centro de Investigación y de Estudios Avanzados, Unidad Mérida, Km. 6 Antigua Carretera a Progreso, Apdo. Postal 73, Mérida, Yucatan 97310, México; c Facultad de Ciencias Básicas, Departamento de Química Física, Universidad de Medellín, Carrera 87 N° 30-65, Medellín 050026, Colombia; d Institut de Química Computacional i Catàlisi and Departament de Química, Universitat de Girona, Maria Aurèlia Capmany 69, Girona, Catalonia 17003, Spain; e Departamento de Química Física, Universidad de Sevilla, c/Profesor García González, s/n, Sevilla 41012, Spain; f Departamento de Química, Universidad del Valle, Calle 13 N° 100−00, Cali 760042, Colombia

## Abstract

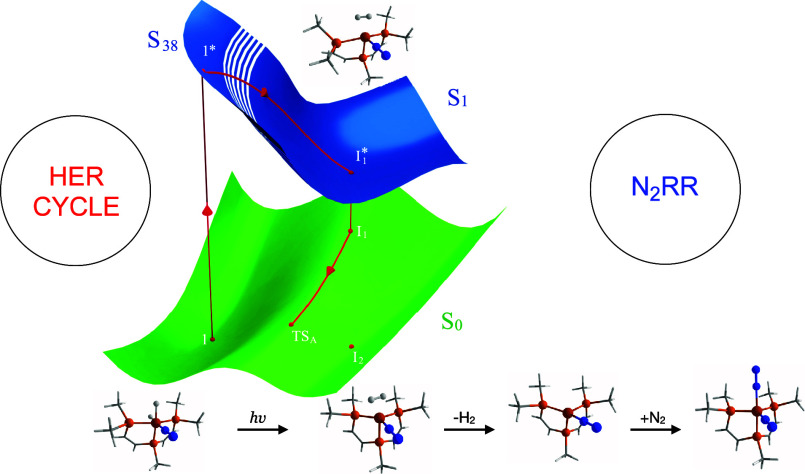

The P_2_P^Ph^Fe(N_2_)(H)_2_ catalyst showed a significant ammonia yield under light irradiation. However, under thermal conditions, the hydrogen evolution reaction (HER) is favored over the nitrogen reduction reaction (N_2_RR), making P_2_P^Ph^Fe(N_2_)(H)_2_ an ideal system for studying the competition between both reactions. In this study, we used a series of computational tools to elucidate the photochemical reaction mechanism for the N_2_RR and thermal pathways leading to the HER with this catalyst. We calculated the energy profile for each transformation and estimated the rate constants for each step. Our results, which are consistent with experimental observations, indicate that photoinduced H_2_ elimination from P_2_P^Ph^Fe(N_2_)(H)_2_ promotes the formation of P_2_P^Ph^Fe(N_2_)_2_, which is on-path for N_2_RR. However, this elimination process is kinetically hindered due to high-energy barriers. Furthermore, our calculations reveal enhanced dinitrogen activation upon the conversion of P_2_P^Ph^Fe(N_2_)(H)_2_ to P_2_P^Ph^Fe(N_2_)_2_.

## Abstract 2

The proposed photochemical catalyst transformations from on-path for HER toward on-path for N_2_RR is thermodynamically favorable at room conditions.

## Introduction

The Haber–Bosch process currently dominates industrial ammonia production. This high-temperature and high-pressure process (300–500 °C and 150–200 atm) relies on Fe-based heterogeneous catalysts to convert N_2_ to NH_3_. However, it consumes approximately 1.0% of global energy and contributes 1.2% of annual CO_2_ emissions.^1−4^ These environmental and energy concerns have driven the search for more sustainable alternatives. One promising alternative involves bioinspired transition metal complexes to catalyze the Nitrogen Reduction Reaction (N_2_RR) under milder conditions: N_2_ + 3 H_2_ 2 NH_3_.^5−9^ Biomimetic iron complexes, mimicking the active site of nitrogenases, hold particular interest due to their potential for effective N_2_RR catalysis. While extensive research has explored the use of iron complexes in understanding nitrogenase function,^10−17^ the first application of biomimetic iron catalysts for N_2_RR came in 2013 with Peters et al.’s synthesis of a tris(phosphine)borane iron complex.^18,19^ This pioneering work led to the development of tetradentate P_3_E-Fe (E = B, C, Si) catalysts capable of producing NH_3_ at lower temperatures.^3,6^ However, despite these improvements, the efficiency of these catalysts remains low, limiting their potential as viable alternatives to the Haber–Bosch process.

**Scheme 1:**
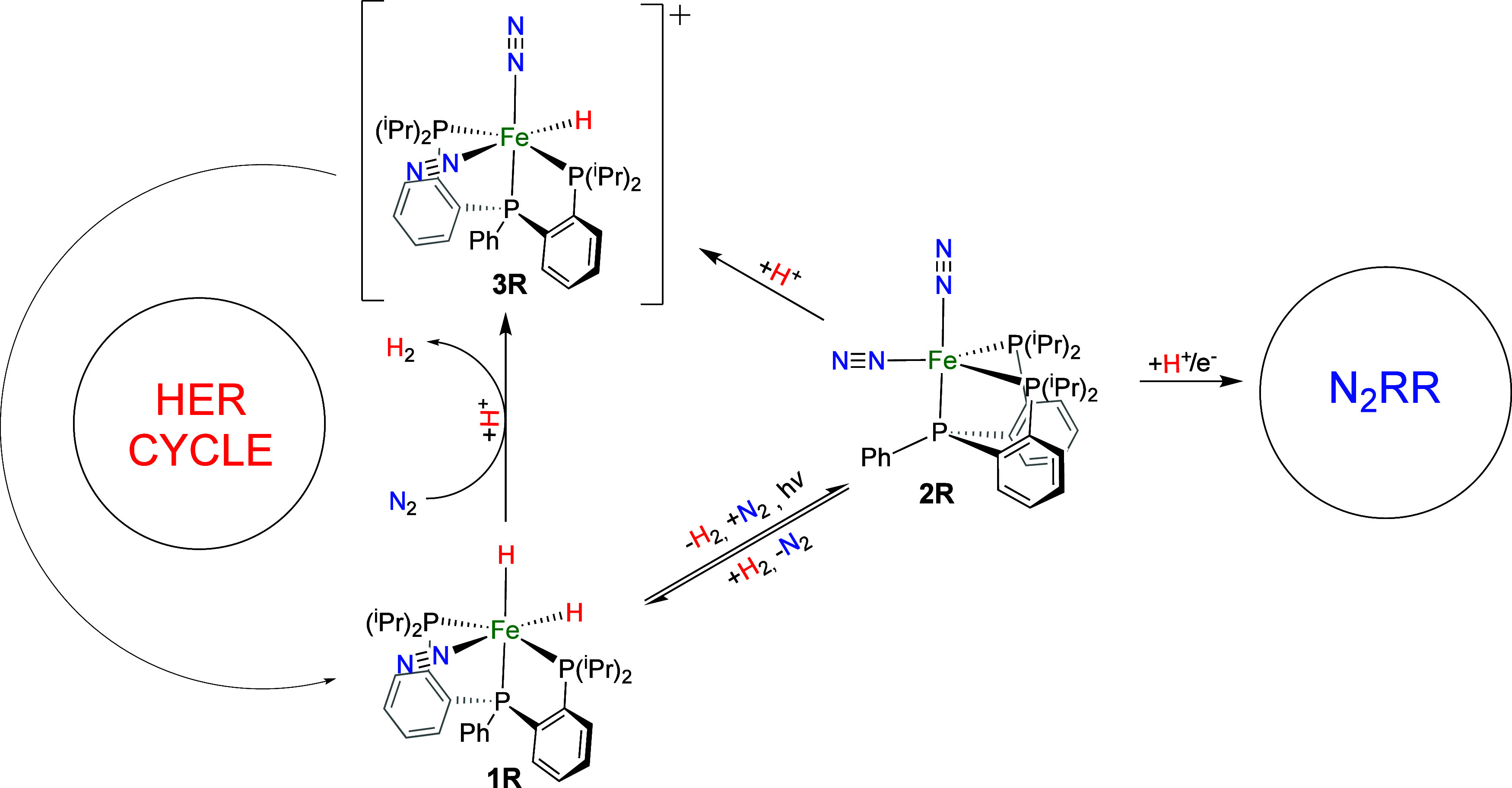
Reactions for Transformations between Catalysts **1R**, **2R**, and **3R**

The low efficiency of iron-based N_2_RR catalysts is attributed to their propensity to catalyze the competing Hydrogen Evolution Reaction (HER).^20,21^ During HER, these catalysts evolve from iron-dinitrogen (Fe-(N_2_)_*x*_) to iron-hydride (Fe-(H)_*x*_) species, ultimately releasing H_2_ through a proton-coupled electron transfer process. Some Fe-(H)_*x*_ species can revert to Fe–N_2_ species via reductive photoelimination of H_2_, followed by the oxidative addition of N_2_.^20,22^ In this context, Schild and Peters^23^ introduced a novel iron-bis(hydride) complex, P_2_P^Ph^Fe(N_2_)(H)_2_ (**1R**) (P_2_P^Ph^ = bis(*o*-diisopropylphosphino-phenyl)-phenylphosphine), exhibiting moderate N_2_RR activity (Scheme 1). Interestingly, irradiation of **1R** under the same reaction conditions significantly enhances the ammonia yield. The authors hypothesized that this photochemical process triggers the substitution of H_2_ in **1R** with N_2_, leading to the formation of P_2_P^Ph^Fe(N_2_)_2_ (**2R**), a key intermediate on-path for the N_2_RR (Scheme 1). This reaction likely reduces the concentration of off-path iron-hydride species for N_2_RR. Furthermore, both **1R** and **2R** can react with a proton source to generate [P_2_P^Ph^Fe(N_2_)_2_(H)]^+^ (**3R**), a complex thought to be crucial for initiating HER (Scheme 1).^23^

The competition between HER and N_2_RR has been the subject of several computational studies.^14,15,17^ DFT calculations on SiP_3_–Fe catalysts, known for their HER activity, revealed that monohydride formation during the HER cycle prevents N_2_RR catalysis.^17^ In addition, bulky ligands on P_3_E-Fe complexes (E = B, C, Si) can hinder H_2_ coordination, precluding hydride formation.^17^ Consequently, these studies found similar binding energies for H_2_ and N_2_ due to their similar coordination modes with the iron center. While previous reports have focused on optimizing catalyst properties and reaction conditions to maximize ammonia yields, the reactivity and electronic structure of species at the intersection of on-path and off-path routes, particularly those involving H_2_ photoelimination, remain largely unexplored.

Given the light-induced transformation of **1R** from an off-path compound to an on-path compound (Scheme 1), **1R** is an exciting system for studying key transformations that promote N_2_RR. There are few systems in the literature capable of undergoing such light-driven transformations. Moreover, no prior studies have explored the thermodynamics and kinetics of the photochemical reaction mechanisms involved in the interconversion of this catalyst. The ability to track N_2_ activation throughout these catalytic transformations is also unprecedented. In this work, we propose reaction mechanisms for (1) photochemical H_2_ elimination from **1R** to generate **2R**, (2) reverse thermal H_2_ addition to **2R**, and (3) protonation of **1R** and **2R** to form complex **3R**. We evaluate the feasibility of these reactions by analyzing thermodynamic (reaction energies) and kinetic (activation energies and rate constants) parameters. Additionally, we assess the degree of dinitrogen activation and the formal oxidation state of different intermediates along the reaction pathway using the effective oxidation state (EOS) analysis.^24^ The results presented here provide valuable insights into the key factors that govern the competition between the N_2_RR and HER from thermodynamic, kinetic, and N_2_ activation perspectives. These insights can guide the design and modification of biomimetic iron complexes to enhance the N_2_RR catalytic efficiency.

## Results and Discussion

### Reaction Mechanisms

**Figure 1:**
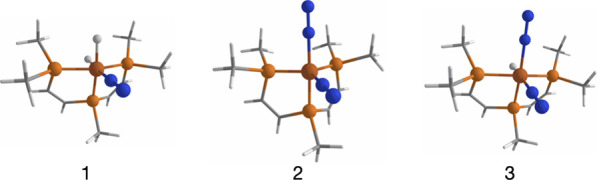
Ball and stick depiction of computational models **1**, **2**, and **3**.

**Scheme 2:**
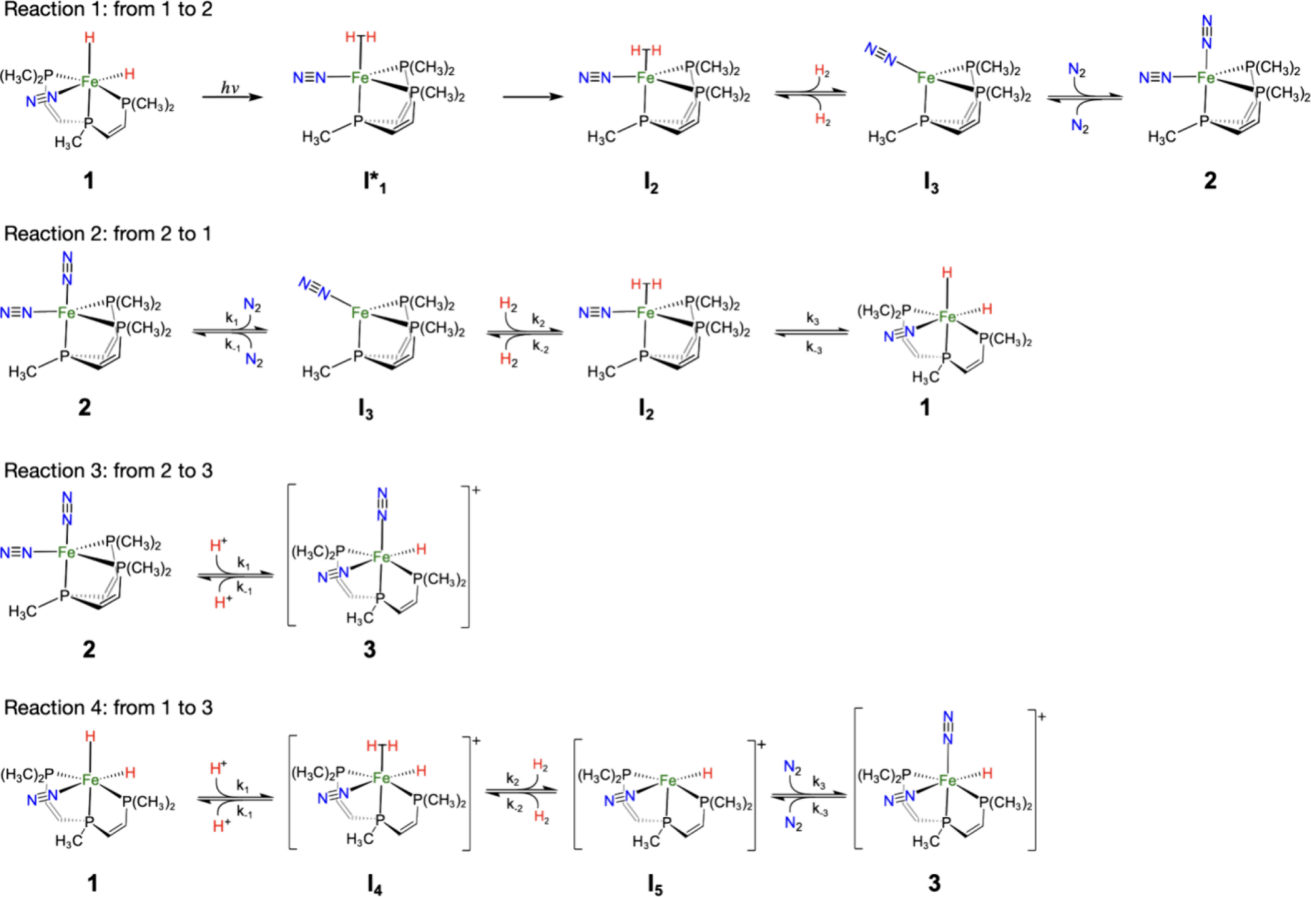
Proposed Reaction Steps for Transformations between Catalysts **1**, **2**, and **3**

DFT calculations were used to propose reaction mechanisms for the photochemical transformations of the catalyst (**1R** → **2R**), the reverse thermal process (**2R** → **1R**), the formation of **3R** from **2R** (**2R** → **3R**), and the direct conversion of **1R** to **3R** (**1R** → **3R**). As described in the Methods section, these reactions were analyzed using models **1**, **2**, and **3**, respectively (Figure 1). Among the different mechanisms explored, we focused on those with the lowest energy barriers for each reaction step. These proposed reactions are labeled 1–4 in Scheme 2. The energy profiles for the transformations among complexes **1**, **2**, and **3** were evaluated using Gibbs free energy in the gas phase at 25 °C and 1 atm. Relative energies were computed with respect to the reactants, considering the reaction stoichiometry (Scheme 2). When diethyl ether, the solvent used in the experiments, was included as an implicit solvent, the energy profiles remained unchanged (Figures S1–S4). The structures of all transition states involved in the studied reactions, including relevant bond lengths, are provided in Figure S5.

#### Photochemical H_2_ Elimination from **1**

We first simulated the absorption spectra of complexes **1**, **2**, and **3** using the TD-DFT approach (Figures S6–S8 and Tables S1–S3). To evaluate the ground state and low-lying excited state energies, we used the PBEh-3c functional,^25,26^ known for its reliability with P_2_P^Ph^Fe complexes.^27^ Despite simplifications in the P_2_P^Ph^ ligand, the calculated UV–vis absorption wavelengths for complexes **1R**, **2R**, and **3R** showed good agreement with the reported values (deviations <50 nm), validating our computational approach.^20,23^ The excited states most likely to be populated, as indicated by the oscillator strengths listed in Tables S1–S3, are associated with intrametal charge transfer (IMCT) and metal–ligand charge transfer (MLCT) transitions.

The transformation from **1R** to **2R** is driven by photons from a mercury lamp (UV–vis light). In this process, dihydride complex **1R** is excited to states that promote the formation of an Fe–H_2_ intermediate through the formation of an intramolecular H–H bond, followed by H_2_ elimination (Scheme 2). To model this light-induced H_2_ elimination from the singlet ground state of complex **1**, we focused on singlet excited states in the range of the UV–vis region with electronic transitions that depopulate the Fe–H σ-orbitals and populate orbitals with contribution from the π*-orbitals of the N_2_ moiety (*d*_N–N_ = 1.103 Å). In complex **1**, these transitions could lead to the lengthening of the Fe–H bond (*d*_Fe–H_ = 1.504 Å), facilitating the H–H bond formation and enhancing N_2_ activation. Excited states of **1** with these characteristics are listed in Table 1. A similar approach was used by Veillard.^28^

**Table 1:**
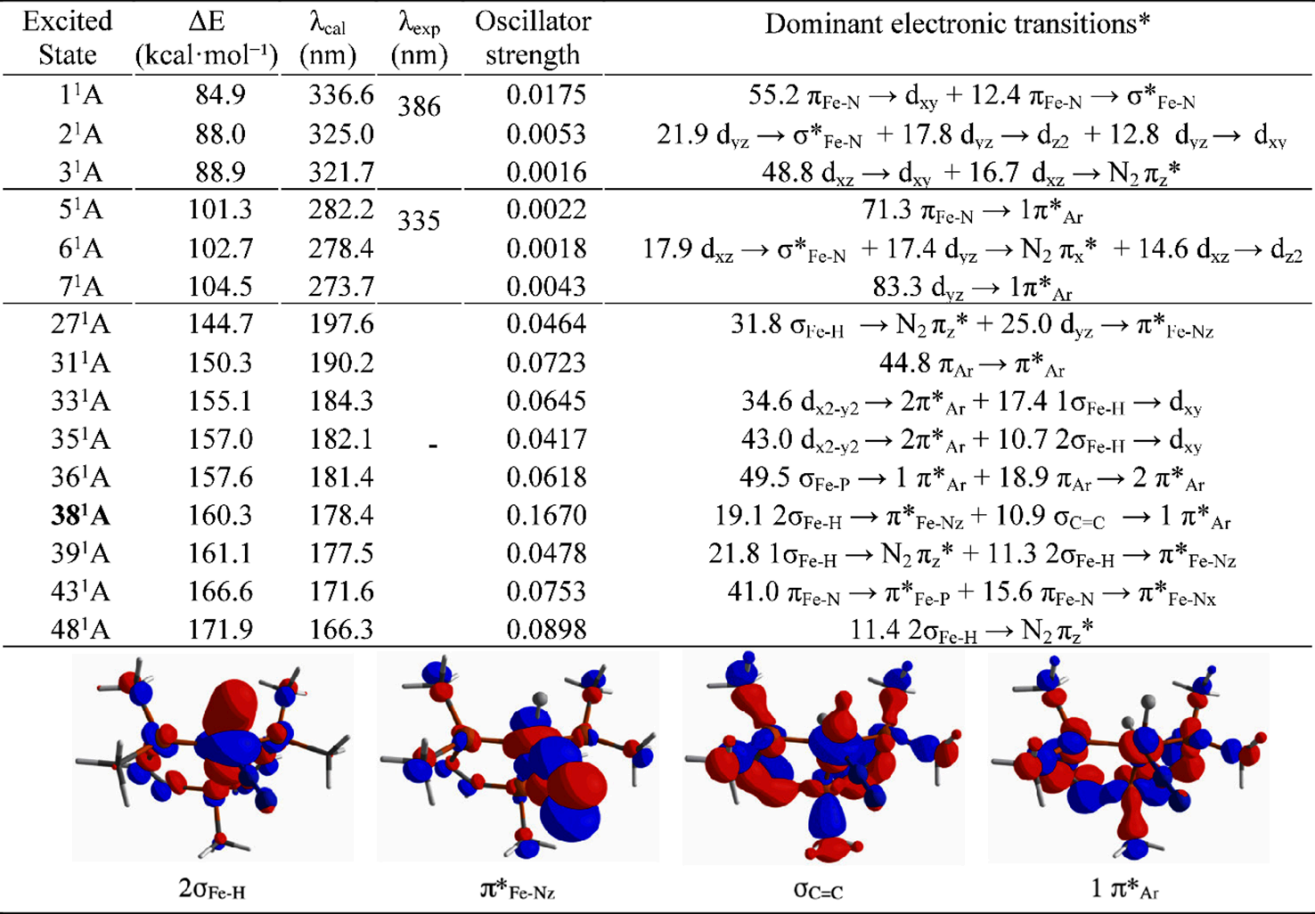
Transition Energies, Wavelength, and Oscillator Strength of the Lowest Excited States of Complex **1**^a^

a Orbitals associated with the dominant transitions that lead to the excited state with the larger oscillator strength (38^1^A) are depicted below. *All involved orbitals are shown in Figure S9.

**Figure 2:**
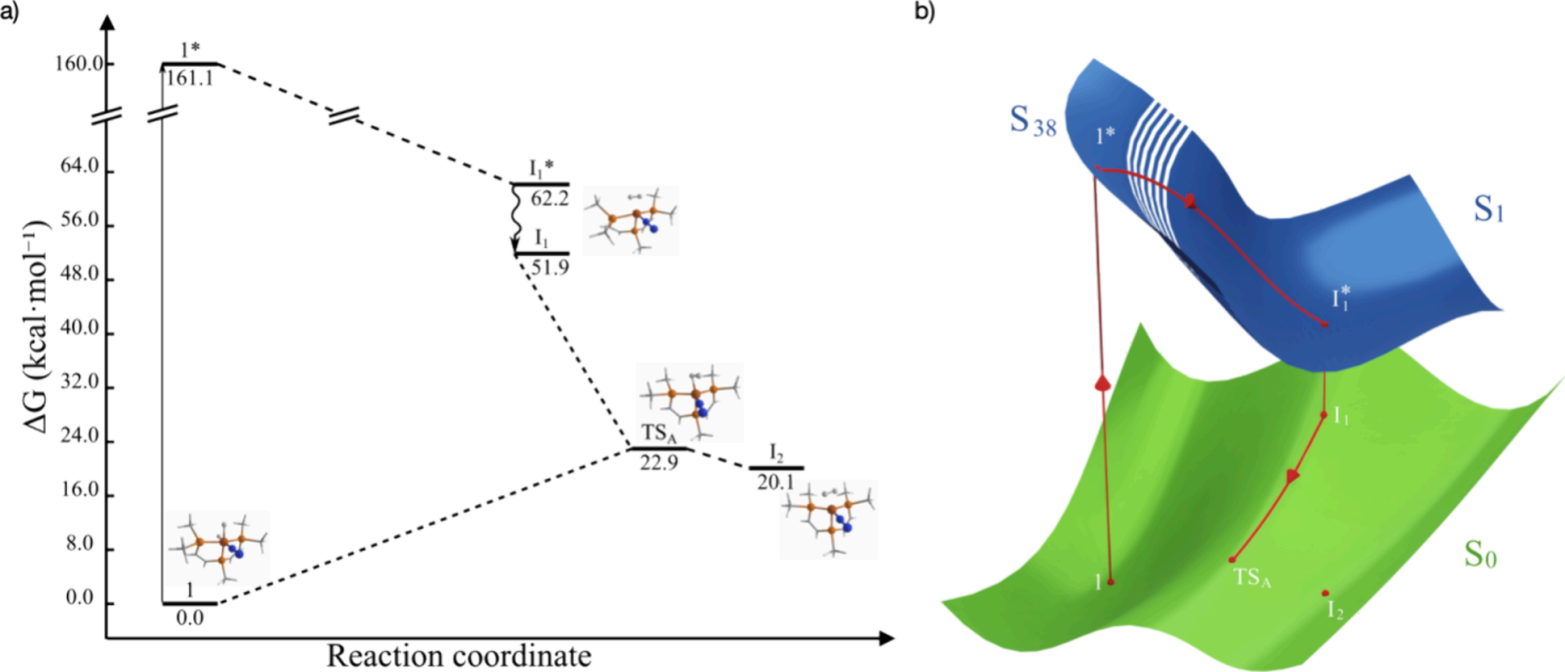
(a) Energy profile of the photochemical conversion from **1** to intermediate **I**_**2**_. (b) Depiction of an avoided crossing between the PESs of the ground state and the first excited singlet state.

After optimizing the geometry of the singlet excited states listed in Table 1 (including crossings between excited states), only 38^1^A converged to a complex resembling Fe(N_2_)(H_2_) (**I**_1_*), featuring side-on H_2_ coordination to the iron center (*d*_Fe–H_ = 1.624 Å and *d*_N–N_ = 1.104 Å; Figure 2a).

Low- and medium-pressure mercury-vapor lamps emit light in the range of 185–600 nm.^29^ The calculated excitation wavelength required to populate state 38^1^A is 178.4 nm. While calculated wavelengths are typically blue-shifted by less than 50 nm relative to experimental values, this estimated wavelength falls slightly outside the emission range of the mercury lamp.

**Figure 3:**
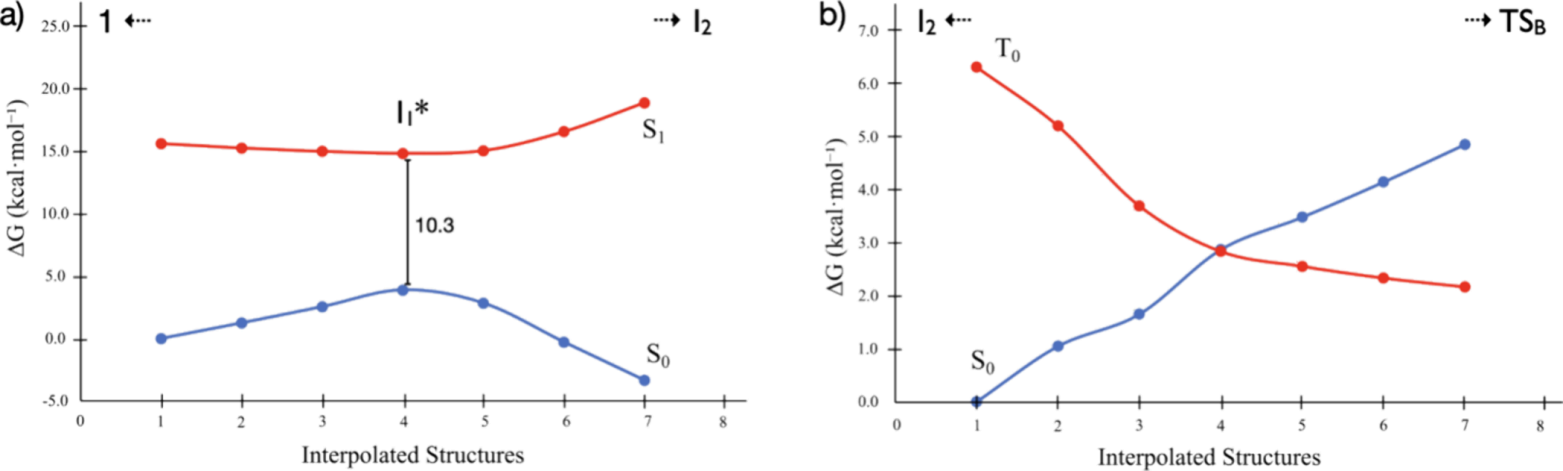
(a) Energy difference of six interpolated structures around intermediate **I**_1_*. (b) Energy difference of six interpolated structures around the MECP between **I**_**2**_ and **TS**_**B**_.

The side-on H_2_ binding represents a classic three centers–two electrons (3c-2e) bond.^30^ Indeed, **I**_1_* is a minimum on the potential energy surface (PES) of the 1^1^A excited state at 62.2 kcal·mol^–1^. This minimum likely arises from nonradiative decay after light absorption by **1**, involving crossings with higher excited state PESs (Figure 2a,b). An intermediate, **I**_**2**_, resembling Fe(N_2_)(H_2_), was located on the singlet state PES at 20.1 kcal·mol^–1^ relative to **1** (*d*_Fe–H_ = 1.542 Å and *d*_N–N_ = 1.108 Å). We anticipated that **I**_1_* would undergo decay to the ground singlet state PES by internal conversion, leading to **I**_**2**_. To explore this conversion, we calculate the energies of the two lowest singlets along 50 interpolated structures between **1** and **I**_**2**_, passing through **I**_1_*. As shown in Figure 3a for six interpolated structures around **I**_1_*, the lowest energy difference occurs at the **I**_1_* geometry (same geometry as **I**_**1**_ at 10.3 kcal·mol^–1^). This energy difference increases as the structure progresses toward **1** or **I**_**2**_ on the reaction pathway, indicative of an avoided crossing.

**Figure 4:**
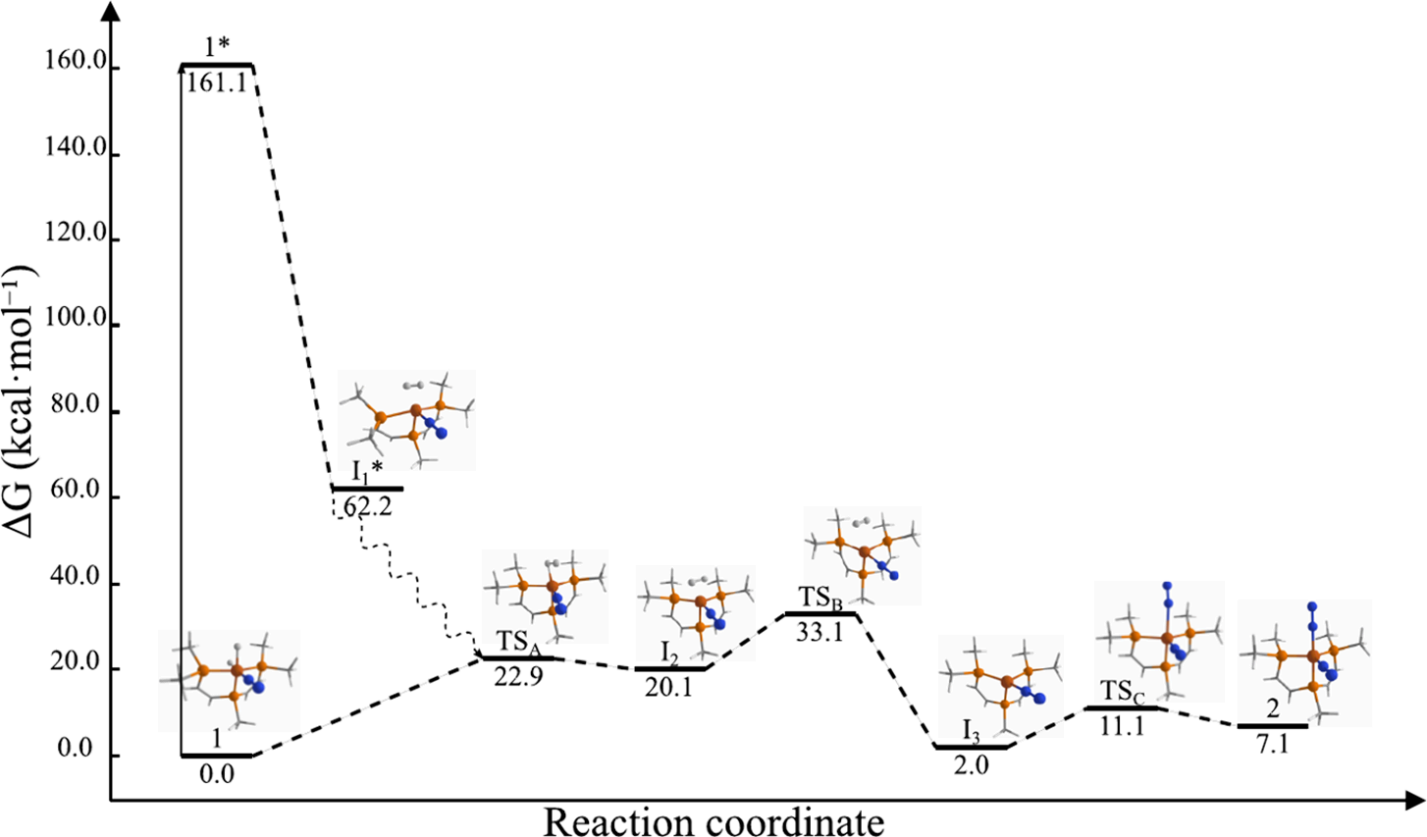
Energy profile of the photochemical transformation from complex **1** to complex **2**.

Further steps in reaction 1 are thermally controlled (Figure 4). After the avoided crossing between **I**_1_* and **I**_**1**_, a saddle point is reached between **I**_**1**_ and **I**_**2**_ at 22.9 kcal·mol^–1^, corresponding to a transition state, **TS**_**A**_ (*d*_Fe–H_ = 1.546 Å and *d*_N–N_ = 1.108 Å). **TS**_**A**_ enables the reaction to proceed either toward the formation of **I**_**2**_ (the desired intermediate) or to revert back to complex **1**. The reverse pathway hinders the formation of the on-path species **2**, consequently limiting the rate of N_2_RR. The high relative energy of **TS**_**A**_ compared to complex **1** (22.9 kcal·mol^–1^) disfavors the thermal formation of complex **2** (on-path for N_2_RR), explaining the low ammonia yield observed at room reaction in the absence of UV–vis light.

The next step is the elimination of H_2_ from **I**_**2**_, leading to the formation of the triplet state intermediate, **I**_**3**_ (*d*_N–N_ = 1.108 Å), at 2.0 kcal·mol^–1^ with respect to reactants (the singlet state is 21.3 kcal·mol^–1^ higher). This exergonic step proceeds through a triplet state transition state, **TS**_**B**_, at a free energy of 33.1 kcal·mol^–1^ (Figure 4). **TS**_**B**_ facilitates cleavage of the Fe–H_2_ bond (*d*_Fe–H_ = 2.413 Å). The four-coordinate **I**_**3**_ species has been proposed as a stable intermediate in similar elimination reactions, as reported in various experimental studies,^20,23^ as well as by some of us.^27,31^

As depicted in Figure 3b, the change in multiplicity from **I**_**2**_ to **TS**_**B**_ involves an intersystem crossing, with the in-between minimum energy crossing point (MECP) located at 28.0 kcal·mol^–1^ (*d*_Fe–H_ = 1.760 Å and *d*_N–N_ = 1.107 Å). Our exploratory calculations on 50 structures around the MECP indicate that the singlet–triplet gap increases with opposite signs as the MECP geometry progresses toward **I**_**2**_ or **TS**_**B**_ (Figure 3b shows only a selection of structures). The final step involves N_2_ addition to **I**_**3**_, forming complex **2** (average *d*_N–N_ = 1.107 Å and *d*_Fe–N_ = 1.798 Å) at 7.1 kcal·mol^–1^ relative to those of the reactants. Although complex **2** has a singlet ground state, its formation proceeds through a triplet transition state, **TS**_**C**_, located at 11.1 kcal·mol^–1^ (Figure 4), implying another intersystem crossing from **I**_**3**_ to **TS**_**C**_. In summary, after the photochemical formation of intermediate **I**_**2**_, reaction 1 can continue through a spontaneous pathway. Most **I**_**2**_ molecules return to **1**, where they are excited again by the lamp in a continuous cycle. However, some **I**_**2**_ molecules proceed to form **2**, as supported by the low barrier associated with **TS**_**B**_ (13.0 kcal·mol^–1^ relative to **I**_**2**_) and the relative energy of complex **2** (−13.0 kcal·mol^–1^ relative to **I**_**2**_). Due to the high-energy barrier between **I**_**3**_ and **I**_**2**_, these molecules are unlikely to revert to **1**. Over time, this results in an accumulation of **2** while a significant amount of **1** remains. This picture is consistent with the experimental results of Schilds and Peters during the photochemical reaction between **1** and **2**, monitored by ^31^P NMR.^23^ Even after 30 min of irradiating with an Hg lamp at −78 °C, complete disappearance of **1** was not observed.

#### H_2_ Addition to **2**

Complex **2** can revert to **1** through the N_2_ elimination followed by H_2_ addition, i.e., following the reverse steps as reaction 1. This reaction is thermodynamically favored but kinetically controlled (*vide infra*). Initially, complex **2** released an N_2_ molecule, forming intermediate **I**_**3**_ at −5.1 kcal·mol^–1^. This step has a very low energy barrier (4.0 kcal·mol^–1^) associated with **TS**_**C**_. **I**_**3**_ then coordinates an H_2_ molecule, producing **I**_**2**_ at 13.0 kcal·mol^–1^. This highest energy barrier (Δ*G*_TSB_ = 26.0 kcal·mol^–1^) is associated with the formation of a 3c-2e bond in Fe–H_2_ binding. The relatively high barrier, which is the rate-determining step of the reaction, explains why this reaction is not observed at −78 °C.^23^ However, at room temperature, this barrier allows **2** to revert to complex **1** (which is active for HER), reducing the catalytic efficiency of **2** for the N_2_RR. Finally, **I**_**2**_ undergoes a spontaneous Fe–H_2_ bond reorganization to regenerate dihydride complex **1** at −7.1 kcal·mol^–1^. As shown in Figure 4, this step has a low barrier of 2.8 kcal·mol^–1^ (Δ*G*_TSA_ = 15.8 kcal·mol^–1^ respect to **2**).

#### Protonation of **2**

**Figure 5:**
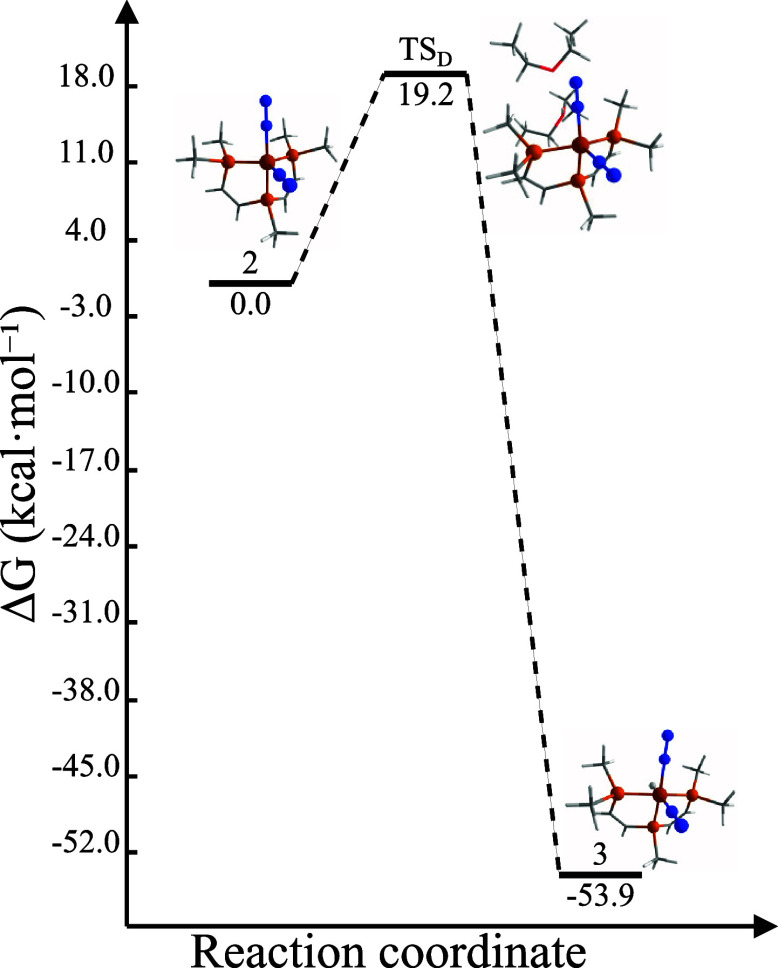
Energy profile of the thermal transformation from **2** to cation **3**.

Complex **2**, an 18-electron species, can also re-enter the HER cycle via acidification with Brookhart acid (HBAr^F^_4_). This reaction (Figure 5) involves a single step: the HBAr^F^_4_ cation protonates the iron center, forming cationic hydride **3** at −53.9 kcal·mol^–1^ with respect to **2**.

Despite the highly favorable thermodynamics, the activation barrier for this reaction via **TS**_**D**_ (*d*_Fe–H_ = 3.280 Å) is relatively high (19.2 kcal·mol^–1^) due to steric hindrance around the iron center during protonation. This steric hindrance explains why the **2R** → **3R** transformation is experimentally observed only at 25 °C. Furthermore, the strongly exergonic nature of this reaction can be attributed to the geometrical change from a distorted trigonal-bipyramidal complex to a more stable distorted octahedral complex.

#### Proton-Assisted H_2_ Elimination from **1**

**Figure 6:**
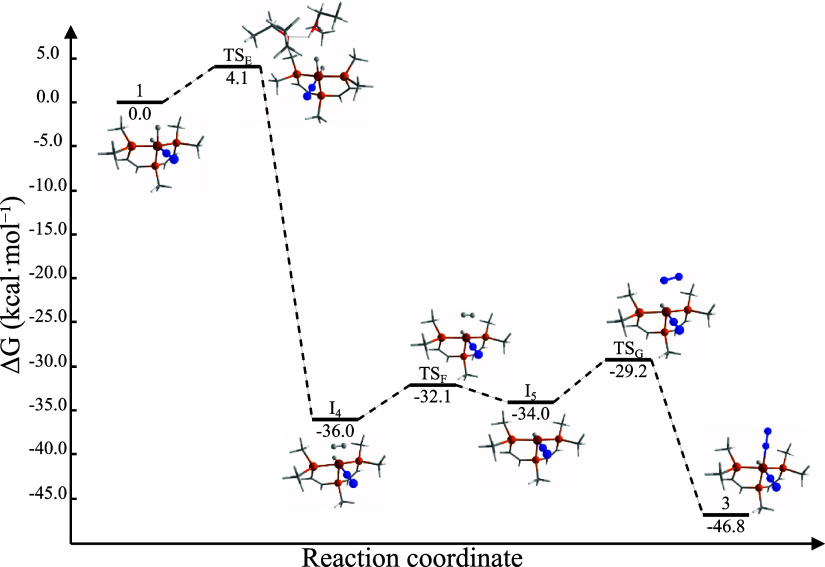
Energy profile of the thermal transformation from **1** to cation **3**.

Complex **1**, a key player in the HER cycle, undergoes a two-step process to form complex **3**. This process involves H_2_ elimination mediated by HBAr^F^_4_, followed by N_2_ addition (Scheme 2). H_2_ elimination begins with donating a proton from HBAr^F^_4_ to one of the hydride ligands in **1**, resulting in the formation and release of an H_2_ molecule. Protonation of hydride complexes with strong acids to generate η^2^-H_2_ complexes is a well-established strategy in the literature.^30,32−36^ Some authors suggest that the proton preferentially attacks the M–H bond rather than the metal center itself.^33,36^ However, in our case, the equatorial hydride remains intact in product **3**, indicating that protonation occurs at the axial hydride ligand in complex **1**. As shown in Figure 6, protonation of the Fe–H_axial_ bond in **1** yields the intermediate [Fe(H_2_)(H)]^+^ (**I**_**4**_), which lies lower in energy than the reactants (**1** + HBAr^F^_4_ + N_2_) by −36.0 kcal·mol^–1^. The relatively high stability of **I**_**4**_ can be attributed to back-donation from the iron 3*d* orbitals to the H_2_ σ∗-orbital of the formed η^2^-Fe–H_2_ species (*d*_Fe–H2_ = 1.583 Å), which can be characterized as a 3c-2e bond.^37^ The formation of **I**_**4**_ occurs with a low energy barrier of only 4.1 kcal·mol^–1^, associated with transition state **TS**_**E**_ (*d*_Fe-Haxial_ = 1.545 Å, Figure 6). This low barrier aligns well with the experimental observation of the **1R** → **3R** conversion at −78 °C, highlighting the ease with which a strong acid like Brookhart acid donates a proton to a hydride ligand, initiating the HER cycle for catalyst **1**. Eliminating the H_2_ ligand from **I**_**4**_ increases the energy to 3.9 kcal·mol^–1^ (with **TS**_**F**_ found at −32.1 kcal·mol^–1^ relative to **1**), leading to the formation of the 16-electron intermediate **I**_**5**_ (Figure 6). **I**_**5**_ is slightly higher in energy than **I**_**4**_, at −34.0 kcal·mol^–1^, reflecting a balance between the increased molecularity (resulting in two molecular fragments) upon H_2_ release, which has a stabilizing entropic effect (Table S4), and the destabilization caused by the decrease in the coordination number of the complex (from 6 to 5).

Finally, the addition of N_2_ leads to the formation of highly stable octahedral complex **3** (−46.8 kcal·mol^–1^). This stability is due to the strong back-bonding interaction between the iron center and N_2_.^38^ As depicted in Figure 6, **3** is formed through the transition state **TS**_**G**_ (−29.2 kcal·mol^–1^), involving end-on coordination of the N_2_ molecule (*d*_Fe–N_ = 2.385 Å). The overall energy profile of the proposed reaction mechanism evidences the thermodynamic favorability and spontaneous nature of the catalyst transformation, supporting its role in HER.

### Reaction Rates

We evaluated the kinetics for each thermal catalyst transformation using the Eyringpy code, which accounts for symmetry effects.^39,40^ The transformations are presented in Scheme 2, with the corresponding rate constants (*k*). These *k* values were estimated at 25 and −78 °C using the transition state theory (Table 2 and Table S5). Minor variations in free energies compared to the energy profiles arise mainly from symmetry constraints in these calculations. The calculations also predict minimal changes in the rate constants due to tunneling corrections for the proton transfer steps (step 1 of reactions 2 and 3). The rate constants, including the Wigner and Eckart tunneling corrections (*k*_eck_), are *k*_1eck_ = 5.8 × 10^10^ s^–1^ and *k*_1eck_ = 2.3 × 10^–21^ cm^3^·molecule^–1^·s^–1^, respectively.

**Table 2:** Calculated Activation (Δ*G*^‡^) and Reaction (Δ*G*) Energies, Forward (*k*_f_) and Reverse (*k*_r_) Rate Constants, and Equilibrium Constant (*K*) for Each Step of the Thermodynamic Catalyst Reaction at 25 °C

parameter	process	**Δ***G^‡^* (kcal·mol^–1^)	**Δ***G*(kcal·mol^–1^)	*k***_f_(s**^**–1**^**)**	*k***_r_(s**^**–1**^**)**	***K***
reaction 2 (**2** → **1**)	step 1	2.8	–5.1	*k*_1_ = 5.7 × 10^10^	a*k*_–1_ = 1.0 × 10^7^	5.47 × 10^3^
	step 2	31.4	18.1	a*k*_2_ = 1.3 × 10^–30^	*k*_–2_ = 2.4 × 10^–17^	5.40 × 10^–14^
	step 3	3.2	–19.6	*k*_3_ = 3.0 × 10^10^	*k*_–3_ = 1.3 × 10^–4^	2.33 × 10^14^
reaction 3 (**2** → **3**)	step 1	19.2	–53.9	a*k*_1_ = 9.2 × 10^–21^	*k*_–1_ = 2.9 × 10^–60^	3.23 × 10^39^
reaction 4 (**1** → **3**)	step 1	3.6	–36.0	a*k*_1_ = 2.5 × 10^–9^	*k*_–1_ = 1.0 × 10^–35^	2.44 × 10^26^
	step 2	3.9	2.0	*k*_2_ = 9.2 × 10^9^	a*k*_–2_ = 2.7 × 10^11^	3.42 × 10^–2^
	step 3	4.3	–13.0	a*k*_3_ = 9.4 × 10^–11^	*k*_–3_ = 2.8 × 10^–20^	3.38 × 10^9^

a Rate constants belonging to bimolecular steps, assumed to be second-order elementary reactions, with units in cm^3^·molecule^–1^·s^–1^.

Table 2 shows that the activation free energies range from 2.8 to 31.4 kcal·mol^–1^. The steps with the highest activation energies are those involving the addition of a fragment to the crowded iron center in reactions 2 and 3 (Δ*G*^‡^ = 31.4 and 19.2 kcal·mol^–1^, respectively). Consequently, these steps are the slowest (*k*_2_ = 1.3 × 10^–30^ cm^3^·molecule^–1^·s^–1^ and *k*_1_ = 9.2 × 10^–21^ cm^3^·molecule^–1^·s^–1^, respectively). In contrast, steps leading to the formation of tetra- or octahedral complexes (which are highly symmetrical) have the lowest free energy values. For instance, step 1 in each reaction has Δ*G* values of −5.1, −53.9, and −36.0 kcal·mol^–1^, respectively. The corresponding equilibrium constants (5.47 × 10^3^, 3.23 × 10^39^, and 2.44 × 10^26^) agree with these reaction energies, indicating thermodynamically favorable steps. The calculated rate constants suggest that converting **1** → **3** is faster than **1** → **2**. Hence, once **1** is formed at room temperature, it preferentially transforms to **3**, promoting the HER cycle. Conversely, light can convert **1** to **2**, which is active for N_2_RR, but it initially forms **I**_**2**_ (see Figure 4). **I**_**2**_ preferably converts to **1** (*k*_3_ = 3.0 × 10^10^ s^–1^) than to **I**_**3**_ (*k*_–2_ = 2.4 × 10^–17^ s^–1^). Furthermore, **2** can be slowly protonated at room temperature to form **3** (*k*_1_ = 9.2 × 10^–21^ cm^3^·molecule^–1^·s^–1^). These kinetic profiles explain the relatively low ammonia yield observed when catalyst **1** is used under light irradiation.^23^

### Dinitrogen Activation

As discussed in the Reaction Mechanisms section, the N–N bond lengths in the various reaction intermediates show minimal variations, making this parameter unsuitable for assessing the degree of dinitrogen activation. Instead, we used Effective Fragment Orbitals (EFOs), derived from the APOST-3D code,^41^ to evaluate the extent of N_2_ activation across different structures. EFOs can be understood as the natural orbitals of individual fragments (e.g., metal or ligand) within a molecule. Core and inner-shell EFOs typically have occupations close to 2, while valence EFOs involved in bonding between fragments exhibit fractional occupations. In the N_2_ unit within the complex, the EFOs resemble those of free N_2_ but show polarization and fractional occupations due to the chemical environment. The occupation numbers of the two π*-type EFOs of N_2_ provide a quantitative diagnostic of N_2_ activation at the stationary points along the proposed catalyst transformations.

Figure S10 depicts the shapes of these EFOs for a model of complex **1**. We focused on a specific N_2_ ligand that remains conserved throughout the catalyst transformations (i.e., the equatorial ligand in species **2** and the one *trans* to the hydride in species **3**). In addition, by analyzing the EFOs of the metal and ligands, we were able to assign oxidation states using the EOS procedure,^24^ also implemented in APOST-3D.^41^ This method relies on the occupations of frontier EFOs to calculate a reliability index (*R*) for the overall oxidation state assignment. Values of *R* below 50% indicate a higher degree of uncertainty in the oxidation state assignments.

**Figure 7:**
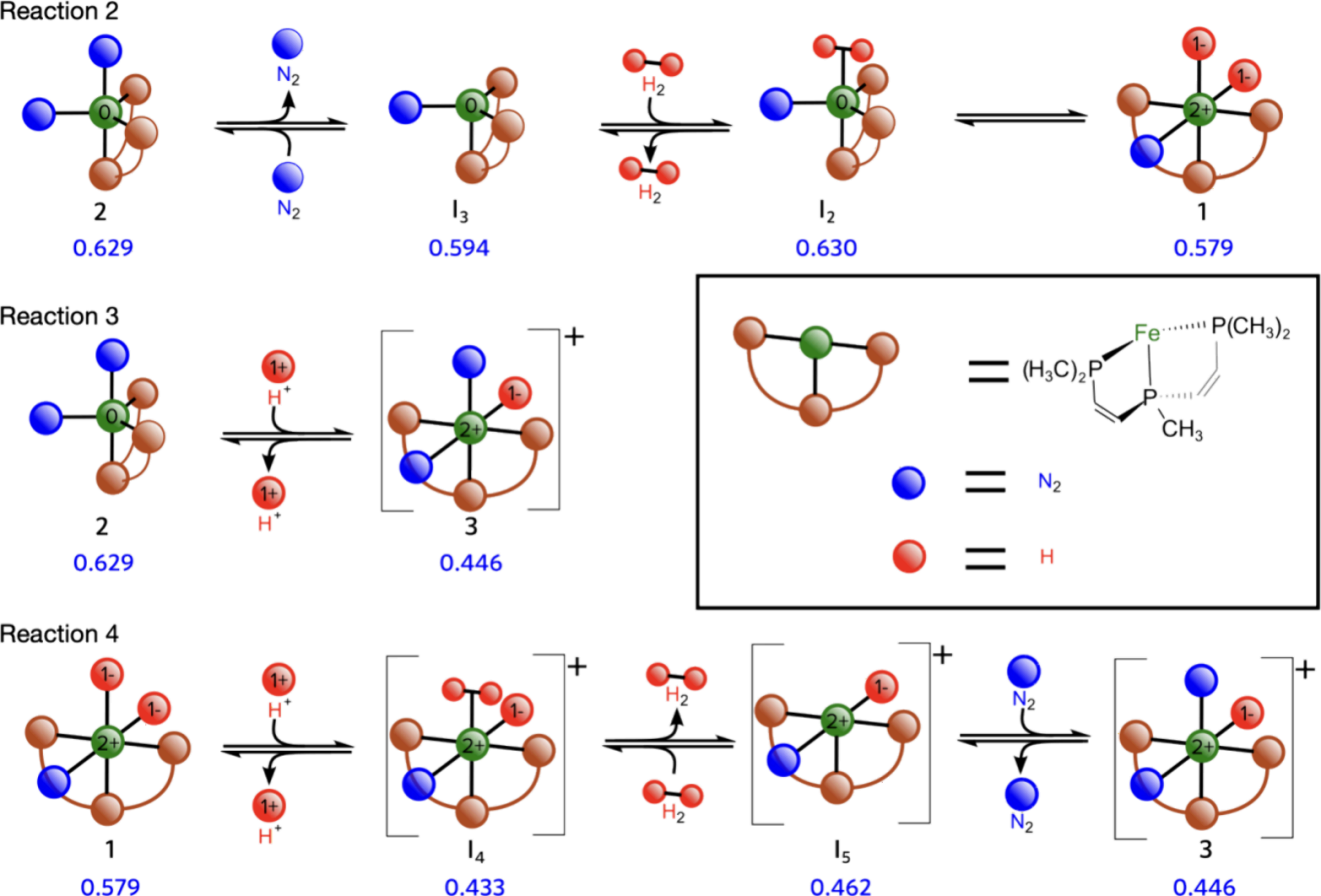
Schematic depiction of complexes involved in the course of reactions 2–4. Values in blue correspond to the sum of the occupations (alpha and beta) of the two π* EFOs of the N_2_ ligand. OS of the metal (green) and proton/hydride (in red).

As shown in Figure 7, species with bipyramidal trigonal geometry exhibit larger occupation of the N_2_ π*-type EFOs, indicating stronger activation of the dinitrogen ligand due to enhanced back-donation from the Fe 3*d* orbitals. The photochemical conversion **1** → **2** significantly increases dinitrogen activation with an EFO occupation of 0.629. This value is consistent with the previous reports by some of us (0.6) based on multiconfigurational wave function analysis.^27^ This suggests that complex **2** is better suited for N_2_RR. While N_2_ activation in complex **2** remains incomplete, it surpasses that in **1** (Figure 7), making complex 2 a more favorable candidate for N_2_RR. Using the EOS scheme, the formal oxidation state assigned for complex **2** is (P_2_P^Ph^)^0^Fe^0^(N_2_^0^)_2_, with a reliability index *R* of 85%. This assignation differs from earlier work by some of us, ((P_2_P^Ph^)^1–^Fe^2+^-[(N_2_)_2_]^1–^),^27^ probably due to the single reference nature of the current EOS analysis. However, both approaches agree on notable activation of the N_2_ molecule, as indicated by the population of the π* orbitals. In contrast, complex **1** is formulated as (P_2_P^Ph^)^0^Fe^2+^(N_2_)^0^(H^1–^)_2_, a structure that persists throughout reaction 4. The assigned OS for complex **1** has moderate reliability (*R*(%) = 62), likely due to the strong electron donation from the hydride ligand. Although the N_2_ ligand shows significant activation, with a π*-type EFOs occupation of 0.579, comparable to that activated molecule CO in TM complexes,^42^ it is still insufficient to consider the N_2_ ligand formally reduced to N_2_^–^. As the catalyst transforms toward the species on-path in the HER pathway, the degree of dinitrogen activation decreases. This is reflected in a low EFO occupation (0.446 for species **3**), which is best described as (P_2_P^Ph^)^0^Fe^2+^(N_2_^0^)_2_(H^1–^).

## Conclusions

This study proposes reaction mechanisms for the photochemical of H_2_ from dihydride complex **1** to dinitrogen complex **2**, followed by the readdition of H_2_ to regenerate **1**, and the protonation of both **1** and **2** to produce the [(P_2_P^Ph^)Fe(N_2_)_2_(H)]^+^ cation (complex **3**). Complex **2** is predicted to be a key complex on-path for the N_2_RR, while **1** and **3** are considered off-path species that favor the HER.

The calculated energy barriers reveal the critical role of optical activation in achieving an efficient N_2_RR catalysis. The conversion of **1** to **2** is disfavored both kinetically (Δ*G*^‡^ = 33.1 kcal·mol^–1^) and thermodynamically (Δ*G* = 7.1 kcal·mol^–1^), consistent with the low ammonia yields observed in the absence of light. In contrast, the proposed photochemical mechanism provides a more favorable route. Here, **1** first forms intermediate **I**_**2**_ (P_2_P^Ph^FeN_2_H_2_) with a lower activation barrier (20.1 kcal·mol^–1^). This intermediate then undergoes conversion to catalyst **2**, and the remaining steps in this photochemical pathway involve low activation barriers once **I**_**2**_ is formed. This underscores the importance of light in overcoming the high-energy transition state (**TS**_**B**_) that hinders the thermal pathway to **2**, aligning well with the increased ammonia yield observed under light irradiation. The regeneration of complex **1** from **I**_**2**_ is favorable both kinetically and thermodynamically, which may contribute to the inefficiency of the catalyst for N_2_RR. Additionally, the return of complex **2** to either **1** or **3** at room temperature is kinetically slow (k = 1.3 × 10^–30^ and k = 9.2 × 10^–21^ s^–1^, respectively). Finally, the degree of N_2_ activation, as is indicated by the occupation of its π* EFOs, decreases as the catalyst progresses from **2** toward **1** and particularly toward **3**.

As final concluding remarks, we emphasize the following points: (1) transformations involving highly symmetrical intermediates, such as tetrahedral or octahedral complexes, show lower reaction energies, indicating greater thermodynamic favorability; (2) the proposed photochemical pathway toward the N_2_RR product is thermodynamically favorable, although it is hindered kinetically by a high-energy transition state; and (3) species with bipyramidal trigonal geometry and lacking hydride ligands display a higher degree of N_2_ activation, attributable to stronger back-donation of electrons from the Fe 3*d* orbitals and the N_2_ π* orbitals.

## Methods

### Models and Computational Details

To study the behavior of these iron complexes ((P_2_P^Ph^)Fe(N_2_)(H)_2_, **1R**; (P_2_P^Ph^)Fe(N_2_)_2_, **2R**; and [(P_2_P^Ph^)Fe(N_2_)_2_(H)]^+^, **3R**) involved in the nitrogen reduction reaction, we employed simplified models (**1**, **2**, and **3**). These models focus solely on the central structure of the complex and make adjustments to the substituents in the P_2_P^Ph^ ligand. For computational efficiency, complex functional groups on the ligand were replaced with simpler ones. Specifically, bridging groups between the two benzene rings were changed from orthophenylene to 1,2-ethene, and bulkier substituents such as isopropyl and phenyl were swapped for methyl groups (see Figure 1). To validate the simplification done in the models, exploratory TD-DFT calculations were carried out to contrast the 10 low-laying excited states of systems **1**, **2**, and **3** against those of **1R**, **2R**, and **3R**, respectively (Tables S6–S8). For both model and real systems, the obtained wavelengths are comparable with deviations lower than 50 nm respect to the lower energy experimental values, validating the use of the chosen calculation level and the simplified models in the calculations. As the reactions **1R** → **3R** and **2R** → **3R** involve protonation, we explicitly included Brookhart’s acid cation ([(Et_2_O)_2_(H)]^+^) in the calculations, mirroring the experimental conditions.

Geometry optimization and single-point energy calculations were done using the ORCA 5.0.3 package.^43,44^ The geometries of **1**, **2**, **3**, intermediates, and transition states were fully optimized with no symmetry constrains using the PBEh-3c hybrid density functional.^25,26^ This is a global hybrid variant of the Perdew–Burke–Ernzerhof (PBE) functional with a relatively large amount of nonlocal Fock-exchange (42%) and correction to the basis set superposition errors (BSSE) and to the London dispersion effects by the gCP and D3 schemes, respectively, which has shown great performance in the description of the geometry of transition metal complexes and is well suited to describe the energies of noncovalent complexes.^45^ PBEh-3c has proven to predict reliable geometries and relative energies in these kinds of systems.^27^ As a test, we have also evaluated the performance of PBEh-3c, in front of CASSCF estimations, in the calculations of the relative stability of the different spin states, namely, the S-T gap (Table S9); the obtained values are comparable in sign and amplitude. The effects of diethyl ether as an implicit solvent (ε = 4.2400 and *n*_D_ = 1.3526) on the Gibbs free energy were included using the CPCM approach.^46^ After geometry optimization and single-point calculations in all stationary points, analytical frequency calculations were performed on the resulting structures at the same level of theory. No imaginary frequencies were found, indicating a local minimum on the potential energy surface (PES). Only one imaginary frequency was found for the transition states, consistent with a vibrational normal mode along the reaction coordinate. All calculations were done with different spin multiplicities (*S* = 0, 1, 2), selecting the state with the lowest multiplicity for the energy profile evaluation (Table S10). In addition, estimations of rate constants were performed based on the transition state theory, according to , as implemented in the Eyringpy code.^40^ Rate constants have been calculated, including symmetry effects.^39^

Karlsruhe group double ζ basis sets were used throughout the entire study,^47,48^ explicitly the valence-double ζ contraction by the Gaussian atomic orbital basis set def2-mSVP. The resolution of identity (RI) approximation was also included in all calculations using the auxiliary basis sets def2/J and def2-TZVP/C.^49,50^ Relativistic effects were included using the Douglas-Kroll-Hess (DKH) Hamiltonian, alongside the reparametrized basis sets, as implemented in ORCA.^51^ Grimme dispersion correction with Becke and Johnson damping (D3BJ) was also included to better describe van der Waals forces.^52,53^ Vibrational normal modes animation and geometries and orbital plotting were done using the Chemcraft tools.^54^

### Reaction Energy Profile Studies

Time-dependent DFT (TD-DFT) calculations were carried out on the model system **1** (Table 1) to investigate the photochemical transformation from **1R** to **2R**. The energies of the 50 lower excited states of **1** in the Franck–Condon region were obtained. Upon inspection of the excited states with oscillator strength greater than 0.05, we selected those that involve electronic transitions of the types (σ_Fe–H_ → π*_Fe–Nz_) and (σ_Fe–H_ → π_z_*_N2_). Exploratory geometry optimization calculations following the selected excited states showed that populating the 38th state leads to an intermediate (**I**_**1**_) consistent with the photochemical reaction. To assess how the ground state and excited state PESs behave close to the geometry of **I**_**1**_, we have used the image-dependent pair potential (IDPP) interpolation method^55^ to obtain 50 structures between **1** and **I**_**2**_, passing through **I**_**1**_. The three structures closer to **I**_**1**_ toward **1** and the three closers to **I**_**1**_ toward **I**_**2**_ were chosen from the interpolated structures. Thus, TD-DFT calculations for one root were performed on these seven structures (including **I**_**1**_) to determine the energy splitting between the ground and first excited states. In addition, for those reactions where the complexes change multiplicity along the minimum energy path (MEP), for instance, between **I**_**4**_ and **TS**_**F**_, a minimum energy crossing point (MECP) calculation was performed to find the geometry of the species at the minimum energy point over the lowest singlet–triplet seam. The IDPP interpolation method was used to obtain 50 geometries around the MECP. Single-point DFT calculations were carried out on the MECP and six closer structures, three on each side. Accurately describing the MECP between different spin states requires a quantum chemical approach that includes spin–orbit coupling (SOC) and can achieve a state energy precision of approximately 0.5 kcal·mol^–1^. This level of accuracy exceeds the capabilities of current DFT methods (approximately 5.0 kcal·mol^–1^). While SOC might have a minor influence on activation energies, it is unlikely to significantly affect the overall reaction kinetics.

To study the transformation from **2** to **1**, we proposed a mechanism in which the axial N_2_ ligand in **2** is eliminated, forming a tetrahedral complex (P_2_P^Ph^)Fe(N_2_) (**I**_**3**_). Then, the addition of an H_2_ molecule to this unsaturated complex produces (P_2_P^Ph^)Fe(N_2_)(H2) (**I**_**2**_) that, after a homolytic cleavage of the H–H bond, gives way to the formation of **1**. Analogously, the proposed transformation mechanism between **2** and **3** involves a single step in which complex **2** experiences direct protonation of the metal center, forming **3**. Finally, to study the transformations from **1** to **3**, we proposed a mechanism where **1** is protonated at the axial hydride ligand, giving rise to [(P_2_P^Ph^)Fe(N_2_)(H)(H_2_)]^+^ (**I**_**4**_). Then, the resulting H_2_ ligand in the axial position is eliminated, forming the complex [(P_2_P^Ph^)Fe(N_2_)(H)]^+^ (**I**_**5**_). The last step of this transformation is the addition of an N_2_ molecule to **I**_**5**_ at the axial position, forming **3**.

### N_2_ Activation and Oxidation State Evaluation

The effective fragment orbitals (EFOs) and the formal oxidation states were obtained with the APOST-3D code^41^ using the topological fuzzy Voronoi cells (TFVC) real-space atomic partitioning scheme^56^ at the PBEh/def2-SVP level. Individual fragments were defined for Fe, the P_2_P^Ph^ ligand, the H^–^ or H_2_ ligands, and each N_2_ ligand, as appropriate, for complexes **1**, **2**, and **3**, and all intermediates shown in Figure 7. The activation of the dinitrogen ligand was estimated based on the sum of the alpha and beta occupations of the two π*-type EFOs, depicted for complex **1** in Figure S10 of the Supporting Information.

## Supporting Information Available

Energy profiles of reactions 1–4 with solvent effects, structures and geometrical parameters for the transitions states involved in reactions 1–4, simulated UV–vis spectra with DFT approaches of complexes **2** and **3**, orbitals involved in electronic transitions of UV–vis spectra of complexes **1**–**3**, and Cartesian coordinates for the optimized geometries of all considered models (PDF)
