# Supplementary material for: Mechanism of Dinitrogen Photoactivation by P2PPhFe Complexes: Thermodynamic and Kinetic Computational Studies
Source: Inorg Chem. 2024 Oct 23;63(44):21364–74. doi: 10.1021/acs.inorgchem.4c04006 (PMC11539056; doi:10.1021/acs.inorgchem.4c04006)
Supplement: Supplementary file 1 — ic4c04006_si_001.pdf [file ic4c04006_si_001.pdf]

Supporting Information

Mechanism of Dinitrogen Photoactivation by $P_2P^{Ph}Fe$ Complexes: Thermodynamic and Kinetic Computational Studies

Camilo Prada,[a] Eugenia Dzib,[b] Francisco Núñez-Zarur,[c] Pedro Salvador,[d] Gabriel Merino,[b]*

Carmen J. Calzado,[e] Jhon Zapata-Rivera.[f]*

[a] Departamento de Química, Universidad de los Andes, Cra 1 No. 18A – 12, 111711, Bogotá. Colombia.

[b] Departamento de Física Aplicada, Centro de Investigación y de Estudios Avanzados, Unidad Mérida. Km. 6 Antigua Carretera a Progreso, Apdo. Postal 73, 97310, Mérida, Yucatan, México. *Email: gmerino@cinvestav.mx

[c] Facultad de Ciencias Básicas, Universidad de Medellín, Carrera 87 N° 30-65, 050026 Medellín, Colombia. Departamento de Química Física.

[d] Institut de Química Computacional i Catàlisi and Departament de Química, Universitat de Girona, Maria Aurèlia Capmany 69, 17003 Girona, Catalonia, Spain.

[e] Departamento de Química Física. Universidad de Sevilla. c/ Profesor García González, s/n. 41012, Sevilla. Spain.

[f] Departamento de Química, Universidad del Valle, Calle 13 N° 100–00, 760042 Cali, Colombia. *Email: jhon.zapata.rivera@correounivalle.edu.co

KEYWORDS: Dinitrogen Activation, Iron Catalysts, N_2RR, HER, DFT Calculations.

Contents

Figure S1. Energy profile of the photochemical transformation from complex **1** to complex **2** using implicit solvent.

Figure S2. Energy profile of the thermal transformation from **2** to **1** using implicit solvent.

Figure S3. Energy profile of the thermal transformation from **2** to cation **3** using implicit solvent.

Figure S4. Energy profile of the thermal transformation from **1** to cation **3** using implicit solvent.

Figure S5. Structures of all transition states involved in reactions 1-4. Relevant bond lengths are included.

Figure S6. Simulated absorption spectrum via transition electric dipole moment of complex **1**.

Figure S7. Simulated absorption spectrum via transition electric dipole moment of complex **2**.

Figure S8. Simulated absorption spectrum via transition electric dipole moment of complex **3**.

Table S1. Transition energies, wavelength, and oscillator strength of the 50 lowest excited states of complex **1**.

Table S2. Transition energies, wavelength, and oscillator strength of the 50 lowest excited states of complex **2**.

Table S3. Transition energies, wavelength, and oscillator strength of the 50 lowest excited states of complex **3**.

Figure S9. Orbitals involved in the dominant electronic transitions leading to the low-lying excited states of complex **1**.

Figure S10. Shape and occupation of the two π*-type EFOs of N_2 in complex **1**. Contour value of 0.1 a.u.

Table S4. Calculated internal energy (ΔE), enthalpy (ΔH) and free energy (ΔG) for each step of the thermodynamic catalyst transformations at 25 ºC without symmetry effects.

Table S5. Calculated activation ($\Delta G^{\ddagger}$) and reaction (ΔG) energies, forward (k_f) and reverse (k_r) rate-constants, and equilibrium constant (K) for each step of the thermodynamic catalyst reactions at -78 ºC.

Table S6. Transition energies and wavelength, and oscillator strength of the 10 lowest excited states of complex **1** and **1R** against experimental wavelength.

Table S7. Transition energies and wavelength, and oscillator strength of the 10 lowest excited states of complex **2** and **2R** against experimental wavelength.

Table S8. Transition energies and wavelength, and oscillator strength of the 10 lowest excited states of complex **3** and **3R** against experimental wavelength.

Table S9. Singlet-Triplet gap along stationary points of reaction 1 at the PBEh-3c and CASSCF level. Active space selected by Fractional Occupation Density (FOD) analysis.

Table S10. Cartesian coordinates of the optimized PBEh-3c geometries and imaginary frequencies characterizing the transition states.

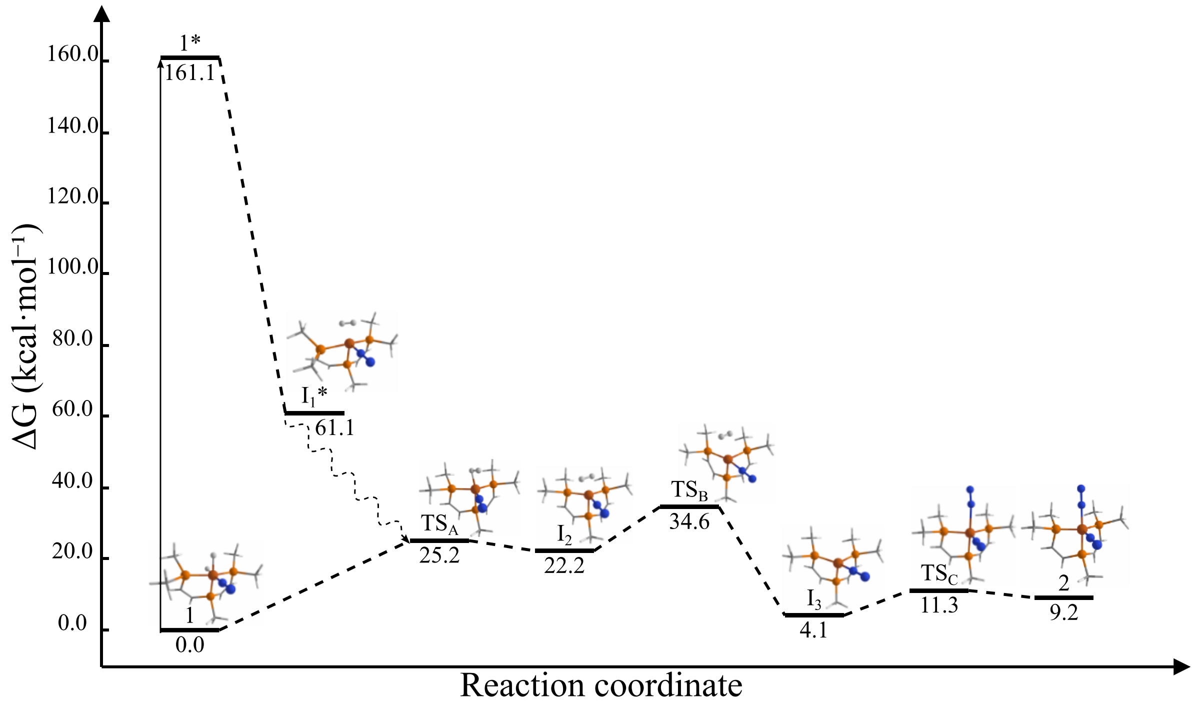

Figure S1. Energy profile of the photochemical transformation from complex **1** to complex **2** using implicit solvent.

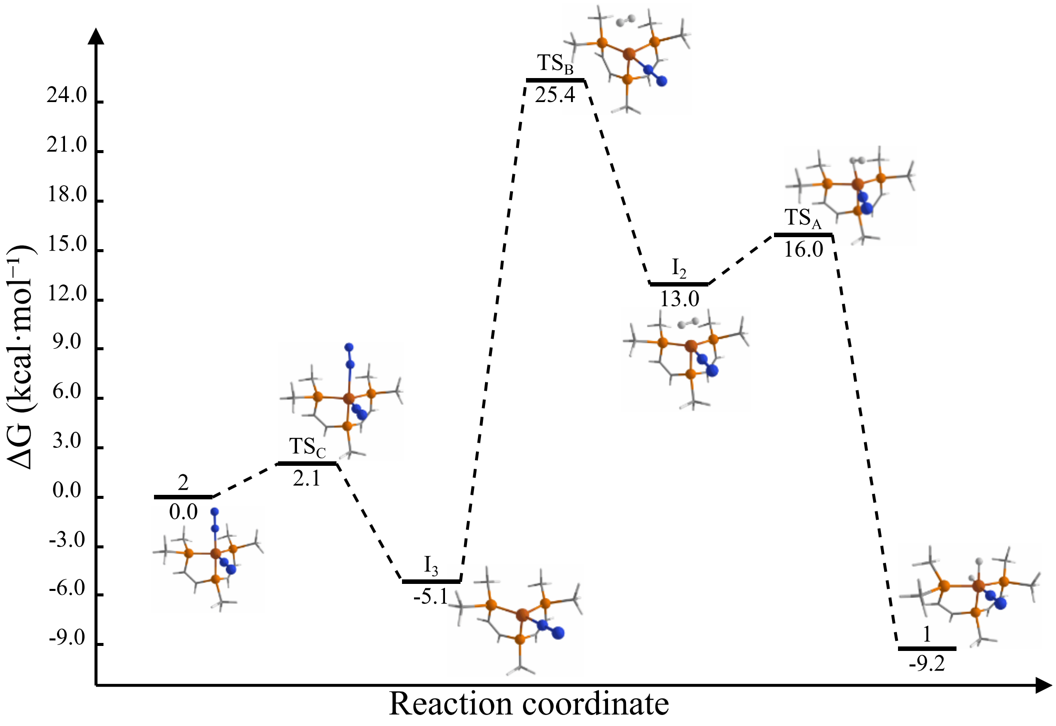

Figure S2. Energy profile of the thermal transformation from **2** to **1** using implicit solvent.

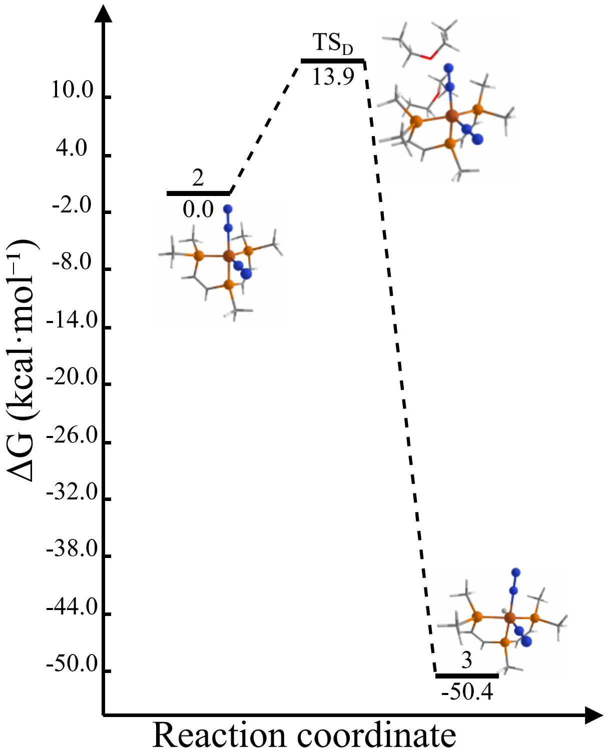

Figure S3. Energy profile of the thermal transformation from **2** to cation **3** using implicit solvent.

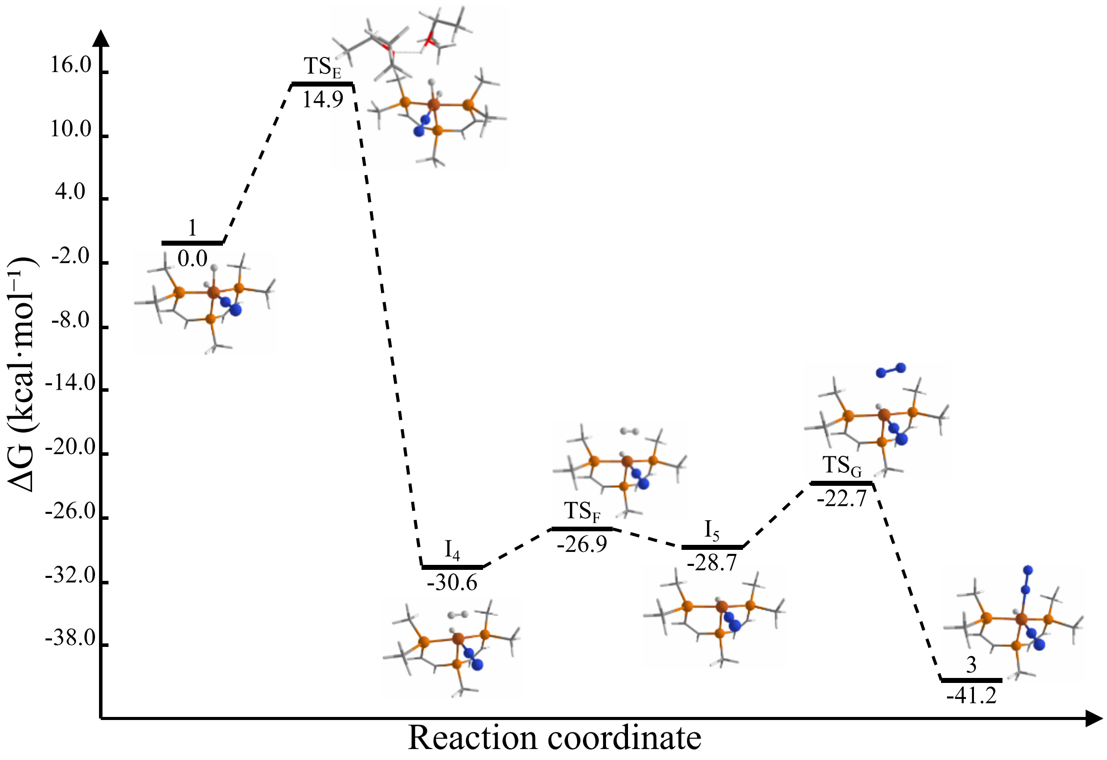

Figure S4. Energy profile of the thermal transformation from **1** to cation **3** using implicit solvent.

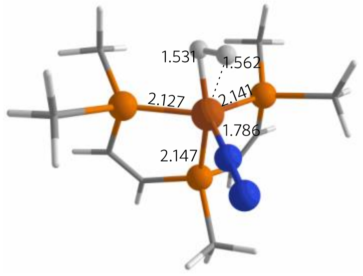

TS$_A$

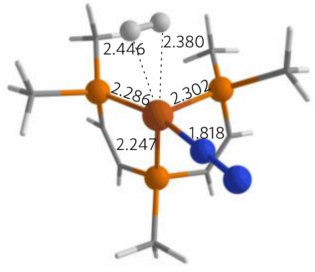

TS$_B$

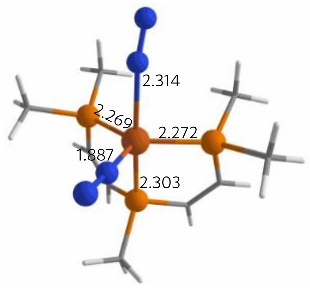

TS$_C$

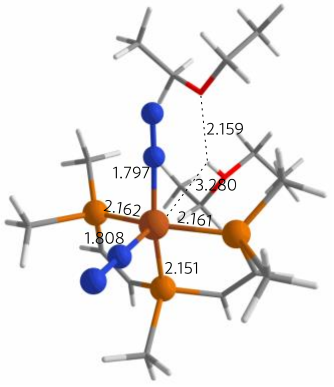

TS$_D$

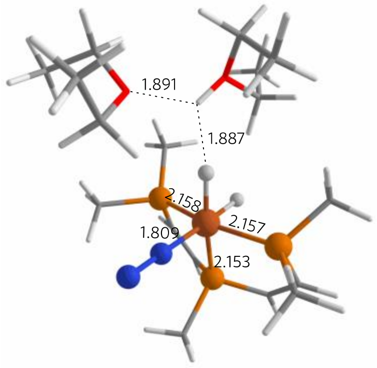

TS$_E$

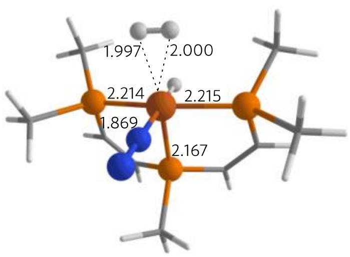

TS$_F$

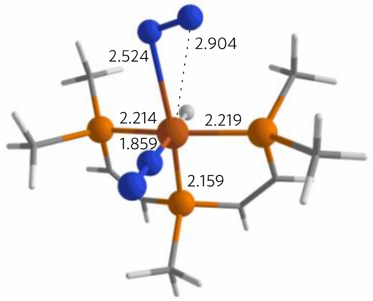

TS$_G$

Figure S5. Structures of all transition states involved in reactions 1-4. Relevant bond lengths are included.

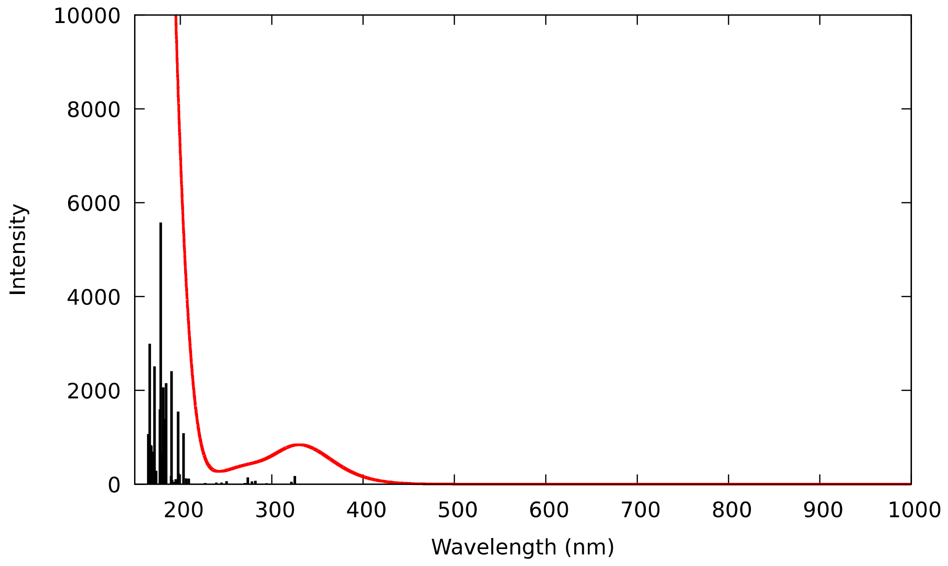

Figure S6. Simulated absorption spectrum via transition electric dipole moment of complex **1**.

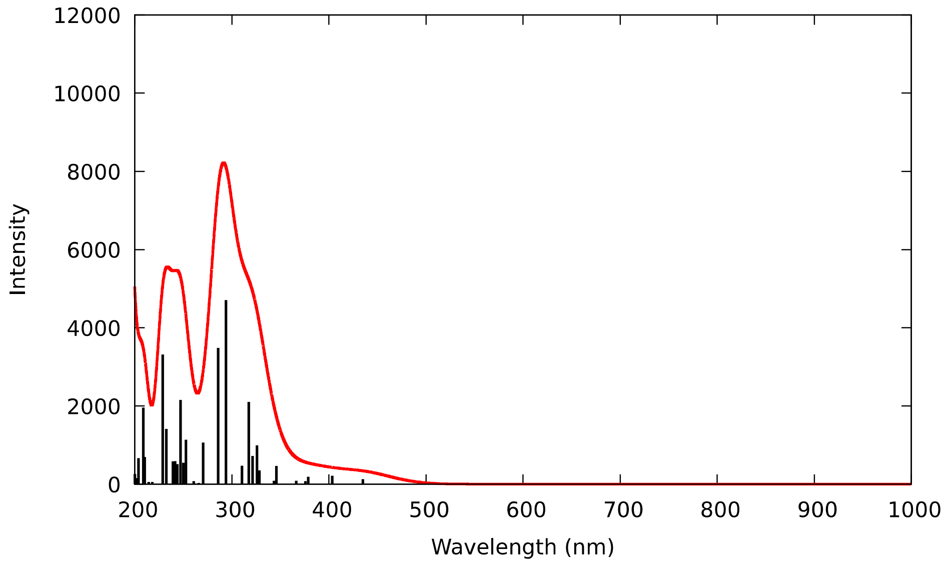

Figure S7. Simulated absorption spectrum via transition electric dipole moment of complex **2**.

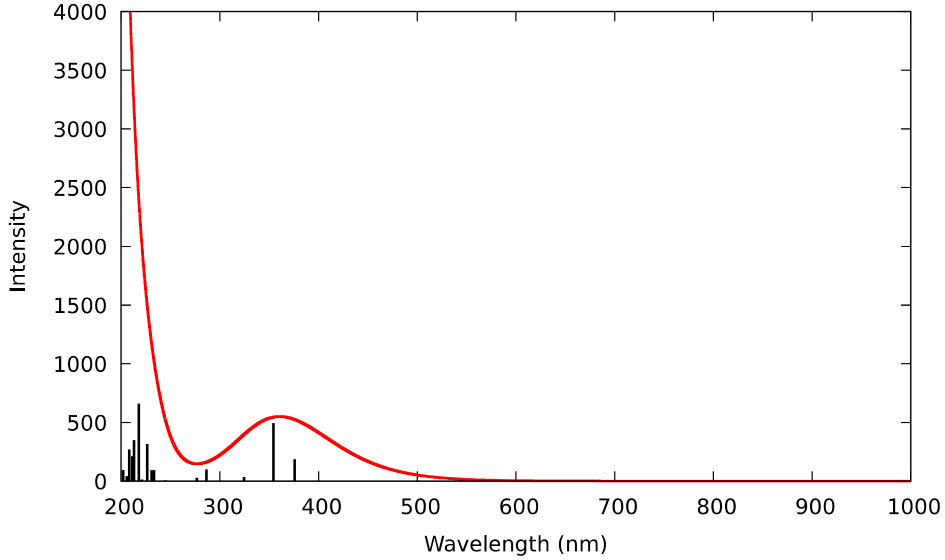

Figure S8. Simulated absorption spectrum via transition electric dipole moment of complex **3**.

Table S1. Transition wavelength and dominant electronic transitions of the 50 lowest excited states of complex **1**.

Excited State	ΔE (kcal/mol)	λ (nm)	λ_{exp} (nm)	f_{osc}	Dominant electronic Transitions (%)
1^1A	84,9	336.6		0,018	(55.2) $\pi_{Fe\text{-}N} \rightarrow d_{xy}$ (12.4) $\pi_{Fe\text{-}N} \rightarrow \sigma^*_{xFe\text{-}H}$
2^1A	88	325		0,005	(21.9) $d_{yz} \rightarrow \sigma^*_{xFe\text{-}H}$ (17.8) $d_{yz} \rightarrow d_{z2}$ (12.8) $d_{yz} \rightarrow d_{xy}$
3^1A	88,9	321.7		0,002	(48.8) $d_{xz} \rightarrow d_{xy}$ (16.7) $d_{xz} \rightarrow \pi^*_{yN\text{-}N}$
4^1A	97,2	294.3	386	0,001	(34.5) $d_{yz} \rightarrow d_{xy}$ (14.1) $d_{yz} \rightarrow \pi^*_{xAr}$ (12.9) $d_{yz} \rightarrow \pi^*_{yN\text{-}N}$
5^1A	101,3	282.2		0,002	(71.3) $\pi_{Fe\text{-}N} \rightarrow \pi^*_{xAr}$
6^1A	102,7	278.4		0,002	(17.9) $d_{xz} \rightarrow \sigma^*_{xFe\text{-}H}$ (17.4) $d_{yz} \rightarrow \pi^*_{xN\text{-}N}$ (14.6) $d_{xz} \rightarrow d_{z2}$
7^1A	104,5	273.7		0,004	(83.3) $d_{yz} \rightarrow \pi^*_{xAr}$
8^1A	105,7	270.6		0,001	(31.5) $\pi_{Fe\text{-}N} \rightarrow \pi^*_{yN\text{-}N}$ (12.8) $\pi_{Fe\text{-}N} \rightarrow \pi^*_{xAr}$ (12.2) $\pi_{Fe\text{-}N} \rightarrow d_{z2}$
9^1A	108,4	263.7		0,000	(84.4) $d_{xz} \rightarrow \pi^*_{xAr}$
10^1A	110,8	258	335	0,000	(37.9) $d_{yz} \rightarrow \pi^*_{xN\text{-}N}$ (23.7) $\pi_{Fe\text{-}N} \rightarrow \pi^*_{Fe\text{-}N}$
11^1A	114,1	250.5		0,002	(48.0) $\pi_{Fe\text{-}N} \rightarrow \pi^*_{xN\text{-}N}$ (24.3) $d_{yz} \rightarrow \pi^*_{Fe\text{-}N}$ (16.0) $d_{yz} \rightarrow \pi^*_{yN\text{-}N}$
12^1A	116,6	245.1		0,001	(90.6) $\pi_{Fe\text{-}N} \rightarrow \pi^*_{yAr}$

13^1A	118,2	241.9		0,000	(78.0) $d_{yz} \rightarrow \pi^*_{yAr}$
14^1A	118,7	240.8		0,000	(24.0) $\pi_{Fe\text{-}N} \rightarrow \pi^*_{Fe\text{-}N}$ (20.1) $d_{yz} \rightarrow \pi^*_{xN\text{-}N}$ (18.9) $d_{yz} \rightarrow \pi^*_{yAr}$ (12.9) $\pi_{Fe\text{-}N} \rightarrow \pi^*_{yN\text{-}N}$
15^1A	119,4	239.4		0,001	(58.2) $\sigma_{xFe\text{-}H} \rightarrow \pi^*_{xAr}$ (33.4) $d_{xz} \rightarrow \pi^*_{yAr}$
16^1A	124,3	230.1		0,000	(36.2) $d_{xz} \rightarrow \pi^*_{xN\text{-}N}$ (28.8) $d_{xz} \rightarrow \pi^*_{yAr}$ (23.9) $\sigma_{xFe\text{-}H} \rightarrow \pi^*_{xAr}$
17^1A	125,9	227.1		0,001	(45.4) $d_{xz} \rightarrow \pi^*_{xN\text{-}N}$ (30.9) $d_{xz} \rightarrow \pi^*_{yAr}$
18^1A	127,7	221.4		0,000	(49.3) $d_{xz} \rightarrow \pi^*_{Fe\text{-}N}$ (27.9) $d_{xz} \rightarrow \pi^*_{yN\text{-}N}$
19^1A	129,1	223.8		0,000	(88.2) $\sigma_{yFe\text{-}H} \rightarrow \pi^*_{xAr}$
20^1A	136,1	210.3		0,000	(61.3) $\sigma_{xFe\text{-}H} \rightarrow \pi^*_{yAr}$ (21.4) $d_{x2\text{-}y2} \rightarrow \pi^*_{xAr}$
21^1A	136,6	209.3		0,000	(56.1) $d_{yz} \rightarrow \pi^*_{yFe\text{-}P}$
22^1A	136,8	209		0,004	(74.7) $\pi_{Fe\text{-}N} \rightarrow \pi^*_{yFe\text{-}P}$
23^1A	138,5	206.4		0,004	(66.6) $\sigma_{Fe\text{-}P} \rightarrow \pi^*_{xAr}$
24^1A	138,7	206.2		0,000	(47.6) $d_{x2\text{-}y2} \rightarrow \pi^*_{xAr}$ (21.3) $\sigma_{xFe\text{-}H} \rightarrow \pi^*_{yAr}$ (14.8) $\sigma_{yFe\text{-}H} \rightarrow \pi^*_{yAr}$ (10.1) $\sigma_{Fe\text{-}P} \rightarrow \pi^*_{yAr}$
25^1A	140,5	203.5		0,033	(34.1) $d_{xz} \rightarrow \pi^*_{yFe\text{-}P}$ (15.5) $\sigma_{Fe\text{-}P} \rightarrow \pi^*_{xAr}$ (13.5) $\sigma_{xFe\text{-}H} \rightarrow d_{xy}$
26^1A	144,5	197.8		0,007	(82.0) $\sigma_{yFe\text{-}H} \rightarrow \pi^*_{yAr}$
27^1A	144,7	197.6		0,046	(31.8) $\sigma_{xFe\text{-}H} \rightarrow \pi^*_{yN\text{-}N}$ (25.0) $d_{yz} \rightarrow \pi^*_{Fe\text{-}N}$
28^1A	146,8	194.8	-	0,003	(24.6) $\sigma_{xFe\text{-}H} \rightarrow d_{xy}$ (19.9) $d_{xz} \rightarrow \pi^*_{yFe\text{-}P}$
29^1A	148,1	193.1		0,001	(29.0) $\pi_{Fe\text{-}N} \rightarrow \pi^*_{Fe\text{-}N}$ (27.5) $\pi_{Fe\text{-}N} \rightarrow \pi^*_{yN\text{-}N}$
30^1A	149.6	191.1		0,072	(71.0) $\sigma_{xFe\text{-}H} \rightarrow \pi^*_{xN\text{-}N}$
31^1A	150,3	190.2		0,072	(44.8) $\pi_{yC\text{-}C} \rightarrow \pi^*_{xAr}$
32^1A	150,6	189.8		0,005	(22.6) $\sigma_{xFe\text{-}H} \rightarrow \pi^*_{yN\text{-}N}$ (22.2) $d_{yz} \rightarrow \pi^*_{Fe\text{-}N}$ (18.1) $\sigma_{xFe\text{-}H} \rightarrow \pi^*_{Fe\text{-}N}$ (14.2) $d_{yz} \rightarrow \pi^*_{yN\text{-}N}$
33^1A	155,1	184.3		0,064	(34.6) $d_{x2\text{-}y2} \rightarrow \pi^*_{yAr}$ (17.4) $\sigma_{yFe\text{-}H} \rightarrow d_{xy}$
34^1A	156,6	182.6		0,009	(35.6) $d_{xz} \rightarrow \pi^*_{yN\text{-}N}$ (24.0) $d_{xz} \rightarrow \pi^*_{Fe\text{-}N}$
35^1A	157	182.1		0,042	(43.0) $d_{x2\text{-}y2} \rightarrow \pi^*_{yAr}$ (10.7) $\sigma_{yFe\text{-}H} \rightarrow d_{xy}$ (10.0) $\sigma_{yFe\text{-}H} \rightarrow \pi^*_{yN\text{-}N}$
36^1A	157,6	181.4		0,062	(49.5) $\sigma_{Fe\text{-}P} \rightarrow \pi^*_{yAr}$ (18.9) $\pi_{yC\text{-}C} \rightarrow \pi^*_{xAr}$
37^1A	158,4	180.5		0,020	(59.2) $\pi_{xC\text{-}C} \rightarrow \pi^*_{xAr}$ (12.3) $\sigma_{xFe\text{-}H} \rightarrow \pi^*_{Fe\text{-}N}$
38^1A	160,3	178.4		0,167	(19.2) $\sigma_{xFe\text{-}H} \rightarrow \pi^*_{Fe\text{-}N}$ (10.9) $\pi_{xC\text{-}C} \rightarrow \pi^*_{xAr}$
39^1A	161,1	177.5		0,048	(21.8) $\sigma_{yFe\text{-}H} \rightarrow \pi^*_{yN\text{-}N}$ (11.3) $\sigma_{xFe\text{-}H} \rightarrow \pi^*_{Fe\text{-}N}$
40^1A	161,2	177.4		0,002	(90.1) $\sigma_{yFe\text{-}H} \rightarrow \pi^*_{xN\text{-}N}$

41^1A	164,8	173.5		0,009	(54.5) σ_{yFe-H} → π^*_{Fe-N} (14.6) σ_{yFe-H} → π^*_{yN-N}
42^1A	165,9	172.3		0,000	(37.2) σ_{xFe-H} → π^*_{yFe-P} (25.5) π_{xC-C} → π^*_{yAr}
43^1A	166,6	171.6		0,075	(41.0) π_{Fe-N} → σ^*_{C-H} (15.7) π_{Fe-N} → π^*_{yFe-P}
44^1A	166,9	171.3		0,019	(21.5) d_{yz} → σ^*_{C-H} (20.0) σ_{xFe-H} → π^*_{yFe-P} (15.3) d_{yz} → π^*_{yFe-P}
45^1A	167,3	170.9		0,021	(18.8) π_{xC-C} → π^*_{yAr} (15.2) d_{yz} → σ^*_{C-H} (13.7) d_{yz} → π^*_{yFe-P} (12.2) σ_{xFe-H} → π^*_{yFe-P} (10.1) σ_{P-C} → π^*_{xAr}
46^1A	170	168.2		0,024	(28.1) π_{yC-C} → π^*_{yAr} (11.2) σ_{C-H} → π^*_{xAr}
47^1A	170,3	167.8		0,025	(33.1) π_{Fe-N} → σ^*_{P-C} (18.04) π_{Fe-N} → d_{z2} (15.2) σ_{yFe-H} → π^*_{yFe-P}
48^1A	171,9	166.3		0,090	(11.4) σ_{xFe-H} → π^*_{yN-N}
49^1A	172,5	165.8		0,000	(41.7) σ_{P-C} → π^*_{xAr} (29.4) π_{xC-C} → π^*_{yAr}
50^1A	173,4	164.9		0,032	(17.6) d_{xz} → π^*_{yFe-P} (14.1) d_{xz} → σ^*_{C-H}

Table S2. Transition wavelength and dominant electronic transitions of the 50 lowest excited states of complex **2**.

Excited State	ΔE (kcal/mol)	λ (nm)	λ_{exp} (nm)	f_{osc}	Dominant electronic Transitions (%)
1^1A	64,0	446.5		0,002	(27.2) π_{xFe-N} → d_{z2} (24.8) π_{xFe-N} → π^*_{xAr} (16.5) π_{xFe-N} → σ^*_{Fe-P} (10.7) π_{xFe-N} → $\pi^*_{P-C^{Ar}}$
2^1A	65,7	434.9	490	0,002	(29.5) d_{xy} → d_{z2} (28.2) d_{xy} → σ^*_{Fe-P} (12.2) d_{xy} → $\pi^*_{P-C^{Ar}}$ (10.2) d_{xy} → π^*_{xAr}
3^1A	67,6	423.2		0,000	(73.4) π_{xFe-N} → π^*_{xAr}
4^1A	70,8	403.6		0,003	(88.8) d_{xy} → π^*_{xAr}
5^1A	75,5	378.7		0,003	(52.6) π_{xFe-N} → $\pi^*_{x\,N_2^{Ax}}$ (22.0) π_{xFe-N} → $\pi^*_{z\,N_2^{Ec}}$
6^1A	76,1	375.8	405	0,001	(34.2) d_{xz} → d_{z2} (22.0) d_{xz} → σ^*_{Fe-P} (13.7) d_{xz} → $\pi^*_{P-C^{Ar}}$
7^1A	78,0	366.5		0,001	(25.2) d_{yz} → d_{z2} (24.7) d_{yz} → σ^*_{Fe-P} (18.8) d_{xy} → $\pi^*_{y\,N_2^{Ax}}$ (10.9) d_{yz} → $\pi^*_{P-C^{Ar}}$
8^1A	82,6	345.9		0,007	(51.8) π_{xFe-N} → $\pi^*_{y\,N_2^{Ax}}$ (30.0) d_{xy} → $\pi^*_{x\,N_2^{Ax}}$
9^1A	83,2	343.4		0,001	(57.3) d_{xy} → $\pi^*_{y\,N_2^{Ax}}$
10^1A	87,0	328.6	322	0,005	(23.3) π_{xFe-N} → $\pi^*_{z\,N_2^{Ec}}$ (21.0) d_{xy} → $\pi^*_{x\,N_2^{Ec}}$ (19.4) π_{xFe-N} → σ^*_{Fe-P} (16.9) π_{xFe-N} → $\pi^*_{x\,N_2^{Ax}}$
11^1A	87,7	325.9		0,014	(77.7) π_{xFe-N} → π^*_{yAr} (13.8) d_{xy} → $\pi^*_{x\,N_2^{Ax}}$

12^1A	89,0	321.4		0,010	(47.8) $d_{xy} \rightarrow \pi^*_{x N_2^{Ec}}$ (16.5) $\pi_{xFe\text{-}N} \rightarrow \pi^*_{z N_2^{Ec}}$ (11.7) $d_{xy} \rightarrow \pi^*_{yAr}$
13^1A	90,0	317.5		0,030	(30.0) $d_{xy} \rightarrow \pi^*_{x N_2^{Ax}}$ (26.1) $\pi_{xFe\text{-}N} \rightarrow \pi^*_{y N_2^{Ax}}$ (17.1) $\pi_{xFe\text{-}N} \rightarrow \pi^*_{yAr}$
14^1A	92,1	310.3		0,007	(82.0) $d_{xy} \rightarrow \pi^*_{yAr}$
15^1A	97,2	294		0,067	(35.0) $\pi_{xFe\text{-}N} \rightarrow \pi^*_{x N_2^{Ec}}$ (17.4) $d_{xy} \rightarrow \sigma^*_{Fe\text{-}P}$
16^1A	100,0	285.9		0,050	(41.5) $\pi_{xFe\text{-}N} \rightarrow \sigma^*_{Fe\text{-}P}$ (15.6) $\pi_{xFe\text{-}N} \rightarrow \pi^*_{z N_2^{Ec}}$ (13.5) $\pi_{xFe\text{-}N} \rightarrow d_{z2}$
17^1A	105,7	270.6		0,015	(46.4) $d_{xy} \rightarrow \pi^*_{z N_2^{Ec}}$ (14.8) $\pi_{xFe\text{-}N} \rightarrow \pi^*_{x N_2^{Ec}}$ (10.4) $d_{xy} \rightarrow \pi^*_{x N_2^{Ax}}$
18^1A	107,5	265.9		0,000	(87.0) $d_{yz} \rightarrow \pi^*_{xAr}$
19^1A	109,4	261.4		0,000	(43.7) $d_{yz} \rightarrow \pi^*_{x N_2^{Ax}}$ (27.0) $d_{xz} \rightarrow \pi^*_{y N_2^{Ax}}$
20^1A	109,7	260.7		0,001	(95.6) $d_{xz} \rightarrow \pi^*_{xAr}$
21^1A	113,1	252.7		0,016	(22.0) $d_{xy} \rightarrow \pi^*_{z N_2^{Ec}}$ (19.5) $d_{xy} \rightarrow \sigma^*_{Fe\text{-}P}$ (12.0) $\pi_{xFe\text{-}N} \rightarrow \pi^*_{x N_2^{Ec}}$
22^1A	114,3	250.2	251	0,008	(29.7) $d_{xy} \rightarrow \pi^*_{xFe\text{-}P}$ (27.0) $d_{yz} \rightarrow \pi^*_{x N_2^{Ax}}$ (23.9) $d_{xz} \rightarrow \pi^*_{y N_2^{Ax}}$
23^1A	115,7	247.1		0,031	(53.9) $\pi_{xFe\text{-}N} \rightarrow \pi^*_{xFe\text{-}P}$
24^1A	117,2	243.9		0,007	(41.7) $d_{xz} \rightarrow \pi^*_{x N_2^{Ax}}$ (35.9) $d_{yz} \rightarrow \pi^*_{y N_2^{Ax}}$
25^1A	118,3	241.6		0,008	(31.9) $d_{xy} \rightarrow \pi^*_{xFe\text{-}P}$ (29.2) $d_{xz} \rightarrow \pi^*_{y N_2^{Ax}}$
26^1A	119,0	240.2		0,000	(48.3) $\pi_{xFe\text{-}N} \rightarrow \pi^*_{zFe\text{-}N}$ (14.6) $d_{xz} \rightarrow \pi\pi^*_{x N_2^{Ec}}$
27^1A	119,4	239.5		0,008	(48.1) $d_{xz} \rightarrow \pi^*_{x N_2^{Ec}}$ (22.2) $\pi_{xFe\text{-}N} \rightarrow \pi^*_{zFe\text{-}N}$
28^1A	122,9	232.6		0,020	(43.4) $d_{yz} \rightarrow \pi^*_{x N_2^{Ec}}$ (28.0) $d_{xy} \rightarrow \pi^*_{zFe\text{-}N}$
29^1A	124,9	228.9		0,047	(41.0) $d_{xy} \rightarrow \pi^*_{zFe\text{-}N}$ (29.8) $d_{yz} \rightarrow \pi^*_{x N_2^{Ec}}$
30^1A	128,2	223.1		0,000	(86.4) $d_{yz} \rightarrow \pi^*_{yAr}$
31^1A	130,6	218.9		0,000	(59.7) $d_{xz} \rightarrow \pi^*_{yAr}$ (13.2) $d_{yz} \rightarrow \sigma^*_{Fe\text{-}P}$
32^1A	131,1	218.1		0,001	(33.6) $d_{xz} \rightarrow \pi^*_{yAr}$ (11.4) $\pi_{xFe\text{-}N} \rightarrow \sigma^*_{x(C\text{-}H)Ar}$ (11.0) $d_{yz} \rightarrow \sigma^*_{Fe\text{-}P}$ (10.8) $\pi_{xFe\text{-}N} \rightarrow \pi^*_{P-C^{Ar}}$
33^1A	132,7	215.5	-	0,000	(23.4) $\pi_{xFe\text{-}N} \rightarrow \sigma^*_{x(C\text{-}H)Ar}$ (20.7) $\pi_{xFe\text{-}N} \rightarrow \pi^*_{P-C^{Ar}}$ (10.3) $d_{yz} \rightarrow \sigma^*_{Fe\text{-}P}$
34^1A	133,4	214.3		0,001	(83.3) $\sigma_{xFe\text{-}P} \rightarrow \pi^*_{xAr}$
35^1A	136,0	210.3		0,010	(23.2) $d_{xy} \rightarrow \sigma^*_{x(C\text{-}H)Ar}$ (20.9) $d_{xy} \rightarrow \pi^*_{P-C^{Ar}}$ (11.2)

					$d_{xy} \rightarrow \pi^*_{yFe-P}$
36^1A	137,0	208.7		0,028	(42.5) $d_{xz} \rightarrow \sigma^*_{Fe-P}$
37^1A	140,2	203.9		0,010	(59.2) $\pi_{xFe-N} \rightarrow \sigma^*_{(C-H)CH_3}$ (27.5) $\pi_{xFe-N} \rightarrow \sigma^*_{y(C-H)Ar}$
38^1A	141,8	201.6		0,002	(64.2) $d_{yz} \rightarrow \pi^*_{z(N-N)ec}$
39^1A	142,8	200.2		0,004	(37.9) $\pi_{Ar} \rightarrow \pi^*_{xAr}$ (14.8) $d_{xy} \rightarrow \sigma^*_{y(C-H)Ar}$ (12.9) $\sigma_{yFe-P} \rightarrow \pi^*_{xAr}$ (10.5) $d_{xy} \rightarrow \sigma^*_{(C-H)CH_3}$
40^1A	144,2	198.3		0,000	(36.2) $\pi_{xFe-N} \rightarrow \pi^*_{(P-C)Ar}$ (31.4) $\pi_{xFe-N} \rightarrow \sigma^*_{x(C-H)Ar}$ (16.5) $\pi_{xFe-N} \rightarrow d_{z2}$
41^1A	144,7	197.5		0,011	(30.1) $\sigma_{yFe-P} \rightarrow \pi^*_{xAr}$ (20.6) $d_{xy} \rightarrow \sigma^*_{(C-H)CH_3}$ (17.1) $d_{xy} \rightarrow \sigma^*_{y(C-H)Ar}$
42^1A	145,9	196		0,005	(39.1) $\pi_{xFe-N} \rightarrow \pi^*_{yFe-P}$ (21.8) $\pi_{xFe-N} \rightarrow \sigma^*_{x(C-H)Ar}$
43^1A	148,1	193		0,016	(51.6) $\pi_{xFe-N} \rightarrow \sigma^*_{y(C-H)Ar}$ (32.9) $\pi_{xFe-N} \rightarrow \sigma^*_{(C-H)CH_3}$
44^1A	148,4	192.7		0,067	(35.1) $d_{xy} \rightarrow \sigma^*_{(C-H)CH_3}$ (24.7) $\pi_{Ar} \rightarrow \pi^*_{xAr}$ (23.1) $\sigma_{yFe-P} \rightarrow \pi^*_{xAr}$
45^1A	149,0	191.9		0,000	(37.6) $d_{xy} \rightarrow \pi^*_{P-C^{Ar}}$ (28.4) $d_{xy} \rightarrow \sigma^*_{x(C-H)Ar}$ (16.2) $d_{xy} \rightarrow d_{z2}$
46^1A	149,3	191.5		0,001	(82.9) $d_{xz} \rightarrow \pi^*_{xFe-P}$
47^1A	151,4	188.8		0,007	(21.7) $d_{xy} \rightarrow \sigma^*_{x(C-H)Ar}$ (18.2) $d_{yz} \rightarrow \pi^*_{xFe-P}$ (13.0) $d_{xy} \rightarrow \pi^*_{yFe-P}$
48^1A	152,3	187.7		0,121	(36.0) $d_{xy} \rightarrow \sigma^*_{y(C-H)Ar}$ (24.6) $d_{xy} \rightarrow \sigma^*_{(C-H)CH_3}$ (12.7) $\sigma_{yFe-P} \rightarrow \pi^*_{xAr}$
49^1A	154,8	184.7		0,018	(26.2) $d_{xy} \rightarrow \pi^*_{yFe-P}$ (25.9) $d_{yz} \rightarrow \pi^*_{xFe-P}$
50^1A	155,4	184		0,052	(38.5) $\pi_{xFe-N} \rightarrow \pi^*_{P-C^{CH_3}}$ (34.1) $\sigma_{xFe-P} \rightarrow \pi^*_{yAr}$

Table S3. Transition wavelength and dominant electronic transitions of the 50 lowest excited states of complex **3**.

Excited State	ΔE (kcal/mol)	λ (nm)	λ_{exp} (nm)	f_{osc}	Dominant electronic Transitions (%)
1^1A	76,0	376.2		0,004	(30.8) $d_{yz} \rightarrow \sigma^*_{P-C}$ (28.0) $d_{xz} \rightarrow \sigma^*_{Fe-H}$
2^1A	76,1	375.8	367	0,004	(31.3) $d_{xz} \rightarrow \sigma^*_{Fe-H}$ (27.7) $d_{yz} \rightarrow \sigma^*_{P-C}$
3^1A	80,7	354.5		0,001	(50.3) $d_{x2-y2} \rightarrow \sigma^*_{Fe-H}$ (11.35) $\sigma_{Fe-C} \rightarrow \sigma^*_{Fe-H}$
4^1A	88,0	324.7		0,001	(43.6) $d_{x2-y2} \rightarrow \sigma^*_{P-C}$

5^1A	99,8	286.5		0,002	(61.3) $d_{yz} \rightarrow \sigma^*_{Fe-H}$ (16.2) $d_{yz} \rightarrow \sigma^*_{P-C}$
6^1A	103,3	276.9		0,001	(44.5) $d_{xz} \rightarrow \sigma^*P\text{-}C$ (11.8) $d_{xz} \rightarrow \sigma^*_{Fe-H}$
7^1A	116,7	245		0,000	(78.7) $d_{x2-y2} \rightarrow \pi^*_{zN_2^{Ec}}$
8^1A	122,5	233.4		0,002	(40.2) $d_{x2-y2} \rightarrow \pi^*_{xN_2^{Ec}}$ (37.7) $d_{yz} \rightarrow \pi^*_{zN_2^{Ec}}$
9^1A	123,8	231		0,002	(88.7) $d_{x2-y2} \rightarrow \pi^*_{yAr}$
10^1A	124,9	228.8		0,000	(34.6) $d_{yz} \rightarrow \pi^*_{xN_2^{Ax}}$ (31.7) $d_{xz} \rightarrow \pi^*_{zN_2^{Ec}}$
11^1A	126,1	226.7		0,007	(55.8) $d_{x2-y2} \rightarrow \pi^*_{xN_2^{Ax}}$
12^1A	129,2	221.3		0,000	(68.6) $d_{yz} \rightarrow \pi^*_{xN_2^{Ec}}$
13^1A	131,1	218.1		0,015	(36.8) $d_{xz} \rightarrow \pi^*_{xN_2^{Ec}}$ (12.2) $d_{yz} \rightarrow \sigma^*_{Fe-P}$
14^1A	131,7	217.1		0,000	(45.3) $d_{yz} \rightarrow \pi^*_{xN_2^{Ax}}$ (31.0) $d_{xz} \rightarrow \pi^*_{zN_2^{Ec}}$
15^1A	134,2	213.1		0,008	(75.0) $d_{yz} \rightarrow \pi^*_{yAr}$
16^1A	134,5	212.5		0,004	(89.1) $d_{xz} \rightarrow \pi^*_{yAr}$
17^1A	135,2	211.4		0,005	(16.6) $d_{x2-y2} \rightarrow \pi^*_{xAr}$ (13.8) $\sigma_{Fe-H} \rightarrow \sigma^*_{Fe-H}$
18^1A	137,2	208.5		0,000	(73.4) $d_{x2-y2} \rightarrow \sigma^*_{Fe-P}$ (11.4) $d_{xz} \rightarrow \pi^*_{zN_2^{Ec}}$
19^1A	137,3	208.3		0,006	(32.3) $d_{xz} \rightarrow \pi^*_{xN_2^{Ax}}$ (23.0) $d_{xz} \rightarrow \pi^*_{xN_2^{Ec}}$ (19.8) $d_{x2-y2} \rightarrow \pi^*_{xAr}$
20^1A	138,9	205.9	-	0,001	(29.2) $d_{z2} \rightarrow \pi^*_{yAr}$ (20.0) $d_{x2-y2} \rightarrow \pi^*_{xAr}$ (11.4) $d_{xz} \rightarrow \pi^*_{xN_2^{Ax}}$
21^1A	141,4	202.2		0,002	(45.7) $d_{z2} \rightarrow \pi^*_{yAr}$ (19.3) $d_{x2-y2} \rightarrow \pi^*_{xAr}$
22^1A	143,2	199.6		0,001	(63.7) $\sigma_{Fe-C} \rightarrow \pi^*_{yAr}$ (18.4) $\pi_{yAr} \rightarrow \pi^*_{yAr}$
23^1A	145,8	196.2		0,008	(44.9) $\sigma_{Fe-H} \rightarrow \pi^*_{yAr}$ (15.5) $\sigma_{Fe-H} \rightarrow \sigma^*_{P-C}$ (12.6) $\sigma_{Fe-H} \rightarrow \sigma^*_{Fe-H}$ (10.1) $\sigma_{Fe-H} \rightarrow \sigma^*_{Fe-H}$
24^1A	147,2	194.2		0,000	(77.6) $d_{xz} \rightarrow \sigma^*_{Fe-P}$ (11.8) $d_{xz} \rightarrow \sigma^*_{P-C}$
25^1A	147,3	194		0,088	(18.8) $\sigma_{Fe-H} \rightarrow \pi^*_{zN_2^{Ec}}$ (14.5) $\sigma_{Fe-H} \rightarrow \sigma^*_{Fe-H}$ (12.1) $d_{yz} \rightarrow \pi^*_{zN_2}{}^{Ec}$
26^1A	148,8	192.1		0,000	(80.0) $d_{yz} \rightarrow \pi^*_{xAr}$
27^1A	149,7	191		0,002	(75.8) $d_{xz} \rightarrow \pi^*_{xAr}$
28^1A	150,0	190.6		0,050	(40.5) $\pi_{yAr} \rightarrow \pi^*_{yAr}$ (21.8) $\sigma_{Fe-C} \rightarrow \pi^*_{yAr}$ (10.4) $\sigma_{P-C} \rightarrow \pi^*_{yAr}$
29^1A	152,1	188		0,000	(39.8) $\sigma_{Fe-H} \rightarrow \pi^*_{xAr}$ (34.9) $\sigma_{Fe-H} \rightarrow \pi^*_{xN_2^{Ec}}$ (11.9) $\sigma_{Fe-H} \rightarrow \pi^*_{xN_2^{Ax}}$

30^1A	152,5	187.5	0,000	(25.9) $\pi_{xAr} \rightarrow \pi^*_{yAr}$ (14.2) $d_{z2} \rightarrow \sigma^*_{Fe-H}$ (11.1) $\sigma_{Fe-H} \rightarrow \pi^*_{z N_2^{Ec}}$
31^1A	155,8	183.5	0,062	(45.2) $\sigma_{Fe-H} \rightarrow \pi^*_{z N_2^{Ec}}$ (12.0) $\pi_{xAr} \rightarrow \pi^*_{yAr}$
32^1A	156,6	182.5	0,088	(13.5) $d_{yz} \rightarrow \sigma^*_{Fe-P}$ (11.7) $\pi_{yAr} \rightarrow \pi^*_{xAr}$
33^1A	157,4	181.6	0,009	(52.8) $d_{z2} \rightarrow \pi^*_{xAr}$ (12.9) $\sigma_{Fe-H} \rightarrow \pi^*_{xAr}$
34^1A	158,3	180.7	0,054	(25.7) $d_{x2-y2} \rightarrow \pi^*_{Fe-P}$ (13.8) $d_{yz} \rightarrow \sigma^*_{Fe-P}$
35^1A	158,9	180	0,001	(72.5) $\sigma_{Fe-H} \rightarrow \pi^*_{x N_2^{Ax}}$
36^1A	159,4	179.3	0,016	(41.3) $\sigma_{Fe-H} \rightarrow \pi^*_{x N_2^{Ec}}$ (35.6) $\sigma_{Fe-H} \rightarrow \pi^*_{xAr}$
37^1A	159,8	178.9	0,009	(28.5) $\sigma_{Fe-C} \rightarrow \pi^*_{xAr}$ (14.9) $\pi_{xAr} \rightarrow \pi^*_{yAr}$
38^1A	162,2	176.3	0,024	(20.5) $d_{z2} \rightarrow \pi^*_{z N_2^{Ec}}$ (19.6) $d_{x2-y2} \rightarrow \pi^*_{Fe-P}$ (11.9) $\sigma_{Fe-C} \rightarrow \pi^*_{xAr}$ (11.3) $d_{z2} \rightarrow \sigma^*_{P-C}$
39^1A	163,4	175	0,036	(26.3) $d_{x2-y2} \rightarrow \pi^*_{Fe-P}$ (12.6) $d_{z2} \rightarrow \sigma^*_{P-C}$ (10.4) $d_{xz} \rightarrow \pi^*_{Fe-P}$
40^1A	163,5	174.9	0,002	(30.2) $\pi_{xAr} \rightarrow \pi^*_{xAr}$ (14.7) $\sigma_{Fe-C} \rightarrow \sigma^*_{Fe-H}$ (13.8) $d_{z2} \rightarrow \pi^*_{xAr}$ (11.1) $\sigma_{P-C} \rightarrow \pi^*_{yAr}$
41^1A	164,6	173.7	0,004	(53.6) $\sigma_{Fe-C} \rightarrow \pi^*_{z N_2^{Ec}}$ (17.2) $\pi_{yAr} \rightarrow \pi^*_{z N_2^{Ec}}$
42^1A	165,5	172.8	0,035	(48.2) $d_{z2} \rightarrow \pi^*_{z N_2^{Ec}}$ (12.1) $d_{z2} \rightarrow \sigma^*_{Fe-H}$
43^1A	166,0	172.3	0,002	(66.4) $d_{z2} \rightarrow \pi^*_{x N_2^{Ec}}$ (11.7) $d_{yz} \rightarrow \pi^*_{Fe-P}$
44^1A	166,8	171.4	0,005	(39.5) $d_{z2} \rightarrow \pi^*_{x N_2^{Ax}}$ (35.9) $d_{yz} \rightarrow \pi^*_{Fe-P}$
45^1A	167,4	170.8	0,033	(14.6) $\sigma_{Fe-C} \rightarrow \sigma^*_{Fe-P}$ (13.3) $\sigma_{Fe-C} \rightarrow \sigma^*_{P-C}$
46^1A	168,1	170.1	0,042	(35.9) $\sigma_{Fe-C} \rightarrow \pi^*_{x N_2^{Ec}}$ (12.2) $\pi_{yAr} \rightarrow \pi^*_{x N_2^{Ec}}$
47^1A	168,8	169.4	0,012	(35.0) $d_{z2} \rightarrow \pi^*_{x N_2^{Ax}}$ (30.9) $d_{yz} \rightarrow \pi^*_{Fe-P}$
48^1A	170,2	168	0,032	(41.9) $\sigma_{Fe-C} \rightarrow \pi^*_{x N_2^{Ax}}$ (14.9) $d_{xz} \rightarrow \pi^*_{Fe-P}$ (11.4) $\pi_{yAr} \rightarrow \pi^*_{x N_2^{Ax}}$
49^1A	170,4	167.8	0,022	(64.5) $d_{x2-y2} \rightarrow \sigma^*_{P-C}$
50^1A	171,0	167.2	0,074	(35.6) $\sigma_{Fe-H} \rightarrow \sigma^*_{Fe-P}$ (21.1) $\sigma_{Fe-H} \rightarrow \sigma^*_{P-C}$

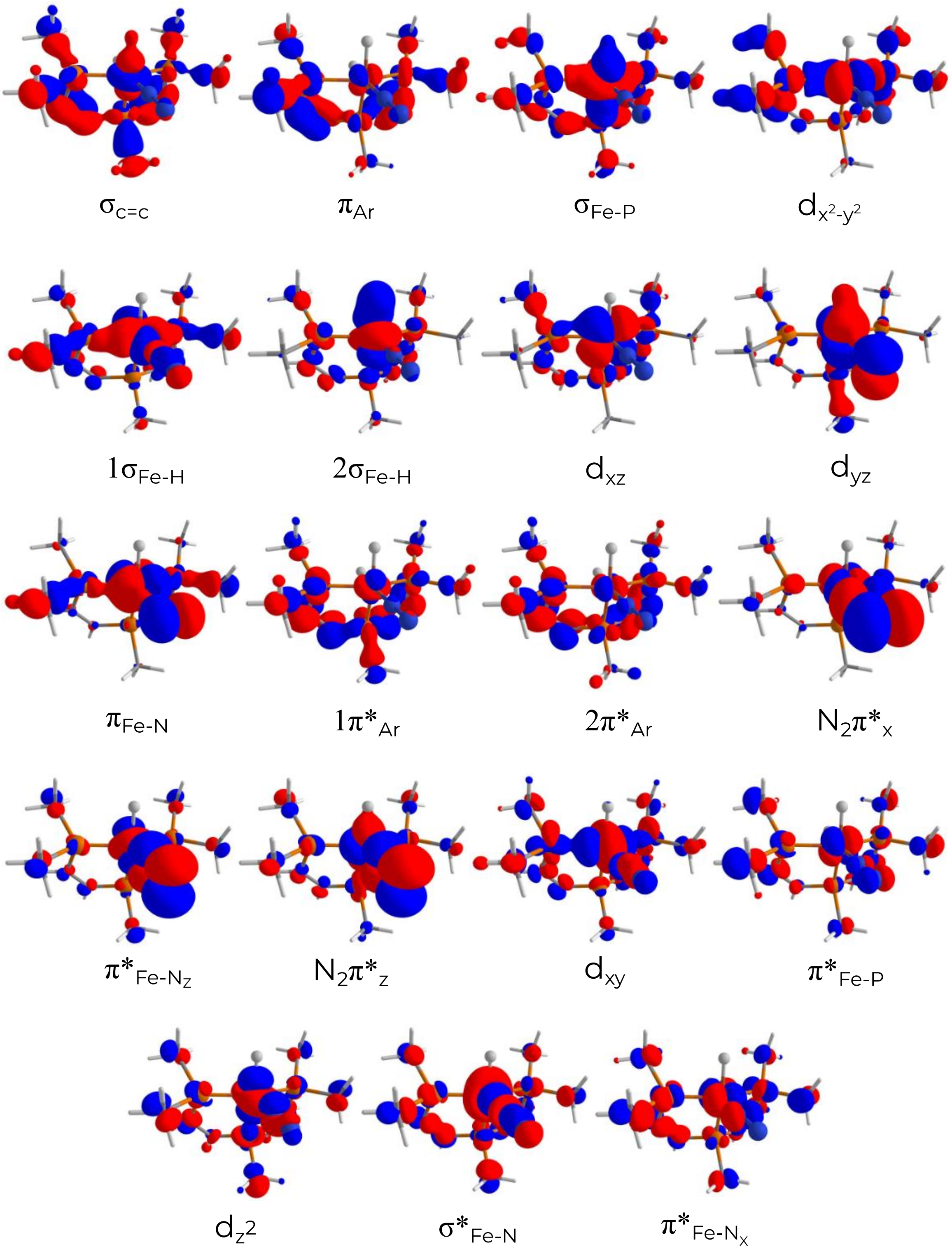

Figure S9. Orbitals involved in the dominant electronic transitions leading to the low-lying excited states of complex **1**.

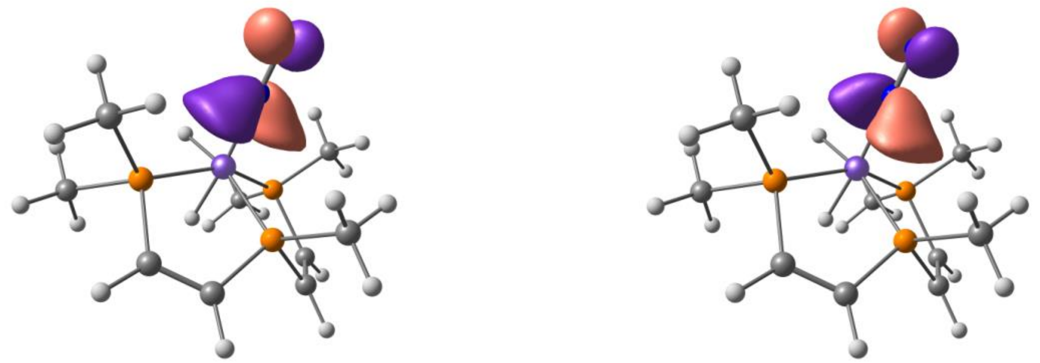

(0.295) **(0.284)**

Figure S10. Shape and occupation of the two π*-type EFOs of N_2 in complex **1**. Contour value of 0.1 a.u.

Table S4. Calculated internal energy (ΔE), enthalpy (ΔH) and free energy (ΔG) for each step of the thermodynamic catalyst transformations at 25 ºC without considering the reaction symmetry.

		**ΔE (kcal·mol^{-1})**	**ΔH (kcal·mol^{-1})**	**ΔG (kcal·mol^{-1})**
Reaction 2 (**2** → **1**)	Step 1	6,9	7,5	-5,1
	Step 2	8,2	7,6	18,1
	Step 3	-20,7	-20,7	-20,1
Reaction 3 (**2** → **3**)	Step 1	-43,5	-42,9	-53,9
Reaction 4 (**1** → **3**)	Step 1	-25,1	-24,5	-36,0
	Step 2	10,5	11,1	2,0
	Step 3	-23,4	-24,0	-12,8

Table S5. Calculated activation (ΔG‡) and reaction (ΔG) energies, forward (k_f) and reverse (k_r) rate-constants, and equilibrium constant (K) for each step of the thermodynamic catalyst reaction at -78 ºC.

		**ΔG‡ (kcal·mol^{-1})**	**ΔG (kcal·mol^{-1})**	**k_f (s^{-1})**	**k_r (s^{-1})**	**K**
Reaction 2 (**2** → **1**)	Step 1	4.5	-0.8	k1=3.9E+07	k-1=4.96E+06	7.87E+00
	Step 2	28.9	14.6	k2=2.1E-40	k-2=4.70E-24	4.46E-17
	Step 3	2.8	-19.9	k3=2.7E+09	k-3=1.40E-13	1.93E+22
Reaction 3 (**2** → **3**)	Step 1	13.7	-58.2	k1=1.9E-22	k-1=1.26E-87	1.50E+65
Reaction 4 (**1** → **3**)	Step 1	-1.7	-38.4	k1=3.6E-05	k-1=3.57E-48	1.01E+43
	Step 2	4.2	5.1	k2=8.1E+07	k-2=4.17E+13	1.94E-06
	Step 3	0.9	-16.8	k3=5.2E-09	k-3=7.98E-28	6.52E+18

Table S6. Transition energies and wavelength, and oscillator strength of the 10 lowest excited states of complex **1** and **1R** against experimental wavelength.

	Real			Model			Experimental
Excited State	ΔE (kcal/mol)	f_{osc}	λ (nm)	ΔE (kcal/mol)	f_{osc}	λ (nm)	λ_{exp} (nm)
1^1A	83.6	0.021	342.0	84.9	0.018	336.6	386
2^1A	84.0	0.020	340.5	88	0.005	325.0	
3^1A	86.1	0.000	332.1	88.9	0.002	321.7	
4^1A	93.3	0.001	324.7	97.2	0.001	294.3	
5^1A	95.1	0.001	306.3	101.3	0.002	282.2	
6^1A	95.7	0.002	300.7	102.7	0.002	278.4	
7^1A	99.4	0.003	298.7	104.5	0.004	273.7	
8^1A	101.1	0.000	282.8	105.7	0.001	270.6	
9^1A	104.2	0.001	274.3	108.4	0.000	263.7	335
10^1A	105.0	0.005	272.3	110.8	0.000	258.0	

Table S7. Transition energies and wavelength, and oscillator strength of the 10 lowest excited states of complex **2** and **2R** against experimental wavelength.

	Real			Model			Experimental
Excited State	ΔE (kcal/mol)	f_{osc}	λ (nm)	ΔE (kcal/mol)	f_{osc}	λ (nm)	λ_{exp} (nm)
1^1A	58.6	0.004	487.8	64.0	0.002	446.5	490
2^1A	63.4	0.000	450.9	65.7	0.002	434.9	
3^1A	66.7	0.032	428.6	67.6	0.000	423.2	
4^1A	69.6	0.003	410.8	70.8	0.003	403.6	
5^1A	71.3	0.006	401.2	75.5	0.003	378.7	405
6^1A	72.0	0.030	397.1	76.1	0.001	375.8	

7^1A	73.2	0.014	390.5	78.0	0.001	366.5	
8^1A	74.3	0.038	385.1	82.6	0.007	345.9	322
9^1A	75.6	0.005	378.4	83.2	0.001	343.4	
10^1A	76.9	0.005	371.6	87.0	0.005	328.6	

Table S8. Transition energies and wavelength, and oscillator strength of the 10 lowest excited states of complex **3** and **3R** against experimental wavelength.

	Real			Model			Experimental
Excited State	ΔE (kcal/mol)	f_{osc}	λ (nm)	ΔE (kcal/mol)	f_{osc}	λ (nm)	λ_{exp} (nm)
1^1A	77.4	0.003	369.5	76.0	0.004	376.2	367
2^1A	78.5	0.008	364.1	76.1	0.004	375.8	
3^1A	82.5	0.014	346.4	80.7	0.001	354.5	
4^1A	86.9	0.003	329.1	88.0	0.001	324.7	
5^1A	102.3	0.004	279.5	99.8	0.002	286.5	
6^1A	102.8	0.001	278.2	103.3	0.001	276.9	
7^1A	108.8	0.000	262.8	116.7	0.000	245.0	-
8^1A	113.9	0.004	251.1	122.5	0.002	233.4	
9^1A	116.0	0.001	246.5	123.8	0.002	231.0	
10^1A	117.0	0.012	244.5	124.9	0.000	228.8	

Table S9. Singlet-Triplet gap along stationary points of reaction 1 at the PBEh-3c and CASSCF level. Active space selected by Fractional Occupation Density (FOD) analysis.

Complexes	1	I2	I3	2
PBEh-3c	-35.0	-42.2	21.3	-24.2
CASSCF	-40.8	-51.7	15.1	-57.1

Table S10. Cartesian coordinates of the optimized PBEh-3c geometries and imaginary frequencies characterizing the transition states.

1, singlet

Fe	1.40358533711824	4.09369271968703	3.46766845632202
P	3.17250409978182	5.23025401986335	3.16184248176420
P	0.33067901534663	2.27162449080337	3.67514369201221
P	2.01851984651843	3.85409797617262	5.49035562782494
C	4.24487651329900	4.83250573043112	1.74682029598669
C	3.19408432652932	7.06079449816237	3.10145712449489
C	3.78496333016509	4.25068828164796	5.62289577320751
C	0.94365521155714	1.43380617948807	5.18385856368445
C	1.35568508470877	4.74322361629958	6.94832992367115
C	1.74797949024395	2.13009267377594	5.99079349026994
C	0.53912480748469	0.97519741363231	2.41555561527949
C	-1.47924386291137	2.19545754578836	3.94464210293160
C	4.28758815069613	4.91654933321688	4.58038370728998
N	0.11662640807159	5.34668656044065	3.56578824619314
N	-0.67277224820766	6.11440498528680	3.62051632801615
H	1.22253376190468	4.00753071141809	1.97536674414679
H	2.44796828415260	3.04576509989559	3.21056825689562
H	1.49786360224423	5.81680114122129	6.80958451909315
H	1.83812531147245	4.44512830725296	7.88245992428895
H	0.28453944705585	4.54871845516161	7.03156580754020
H	0.10333620073833	1.32662936580931	1.47847109227242
H	0.06424912182003	0.03325141142552	2.70117677088128
H	1.60380504409282	0.81288683926675	2.24555159869504
H	3.71445856207898	5.08366407868784	0.82637069764373
H	4.43628353981359	3.75917771392344	1.73692288504915
H	5.19311392534372	5.37495279030644	1.77390784172193
H	2.64929001991753	7.38981067738245	2.21339653878566
H	4.20504925106653	7.47409227645882	3.06377681919799
H	2.68009844100333	7.46436556319609	3.97575953361644
H	-1.98379975508311	2.56900746420066	3.05041348236037
H	-1.75566783602592	2.84381628348605	4.77814961363358
H	-1.83792427652737	1.18360466987660	4.15003602903087
H	5.32196927751257	5.26297127187522	4.57160032087339
H	4.37593488447463	4.02968179427570	6.51121426030785
H	0.65886466238549	0.40596211648568	5.41294005257023
H	2.14932302015722	1.71172594369718	6.91329578244672

2, singlet

Fe	-0.52629473486097	-0.32515668407810	-0.92337466605350
P	-2.38515606869223	-1.14202502220108	-0.25847346959538
P	-0.32720192541029	1.80024987195663	-0.98416965283779
N	-1.38436404510757	-0.41467198307531	-3.69772155959430
N	-1.04701636801690	-0.38863768475444	-2.64512196785577
C	1.02437962660901	2.56775134349360	-1.96046367932651

C	-1.71586455694355	2.87423864814727	-1.51771400742876
C	-2.79424605672613	-2.89647426865440	-0.61180245465121
C	-3.98776910866752	-0.37677594503774	-0.72049427058004
C	0.14612512203133	1.60795303149311	1.66065413720316
C	-2.50972637439744	-1.12702015614575	1.55746139213658
C	-1.45020757355022	-0.66872234073177	2.22276019891399
C	1.36205225919445	-0.93487238647468	1.97894624577948
C	0.02074486264667	2.48555185292147	0.66597309500919
P	-0.06597830170514	-0.12977659889512	1.17851387995401
N	0.88803982492733	-1.38783877970813	-1.22956614526888
N	1.76254022514211	-2.04309534087017	-1.41500929094410
H	0.88305214132238	2.32163881794764	-3.01574341534140
H	1.97916831294872	2.14188375748426	-1.64798529693357
H	1.06477058559838	3.65633154348109	-1.86044433387841
H	-4.07958742951379	-0.36538246941658	-1.80939125136773
H	-4.01135191575263	0.65784254900914	-0.37450339715079
H	-4.84944770705748	-0.90932272123863	-0.30850661130964
H	-2.86960366390753	-3.03158059860285	-1.69354662630134
H	-3.73442211688929	-3.21852695300566	-0.15505720588073
H	-1.98381483213281	-3.53367027613906	-0.25447671614335
H	-3.40774833505503	-1.47759215636169	2.06731955689311
H	-1.41360865742322	-0.61890115449815	3.31077877019168
H	2.28243526954122	-0.60606666722666	1.49280489803662
H	1.28281662340179	-2.01665582409392	1.85630946158128
H	1.42515690064576	-0.70485855920957	3.04497917153285
H	0.12250171915701	3.55983549225524	0.82365939063622
H	0.35896049760851	1.90666917754524	2.68684395499773
H	-1.99631417760879	2.61648982971920	-2.54196290167143
H	-1.46960392633531	3.93924659308622	-1.48606515695758
H	-2.58151609502078	2.68922206187937	-0.87988007579365

3, singlet

Fe	1.49604781423138	4.04168469824649	3.36013338522861
P	3.31099195318558	5.28858286418010	3.18603579687879
P	0.31271052891402	2.20666851799629	3.69391452920775
P	2.10108250580058	3.79694935953039	5.44288848946508
C	4.37807633034905	4.98603854621117	1.75264931122317
C	3.20879310067354	7.10372893349778	3.23792582384702
C	3.84955738956922	4.19423183554231	5.60563345730737
C	0.97133604688235	1.40227687042977	5.17778667363331
C	1.34913469183704	4.75223775575767	6.79684007927009
C	1.80578873975221	2.09531343559784	5.95260364947640
C	0.42053065130604	0.91868154971989	2.42382929336765
C	-1.47179303843963	2.29338358689903	4.03345522211271
C	4.37223949160312	4.89868505960955	4.60180865737751
N	0.20707507343548	5.34807953925994	3.67580844363709
N	-0.54843580002534	6.11535221030952	3.87699932355766
N	1.20409465292980	4.02518439216232	1.53128061602526

H	2.53904369642141	2.99354089433885	3.15772433305157
N	1.04918582777630	3.99747050824570	0.44775316189635
H	2.66181957876114	7.46330438588597	2.36329083588742
H	4.19749365578230	7.56746203200572	3.23866584942139
H	2.67195301530608	7.42966952141067	4.13031189981531
H	3.87424322651862	5.29925182213951	0.83583526983783
H	4.60100823311840	3.92126486298742	1.67461493318753
H	5.31443090809132	5.54254285508668	1.82994390316498
H	-0.06856996755856	1.25263166996833	1.50638112114706
H	-0.06821209538470	-0.00132981518202	2.75125226043106
H	1.46658921719205	0.70740969816905	2.19883767907674
H	-1.99412145582947	2.66605694273934	3.14919077077974
H	-1.66859339616229	2.98131814647922	4.85731277801423
H	-1.88735209002994	1.31619592017694	4.28899059705721
H	0.27521047914197	4.56004947655804	6.83674991034227
H	1.50659093517317	5.81987782269890	6.63196127850481
H	1.78103095554769	4.48732415059474	7.76411711869580
H	5.40240532769189	5.25336208476636	4.62577854400881
H	4.41798309418948	3.94085546873480	6.49897466261825
H	0.66779305623178	0.38574492307626	5.42701525394184
H	2.22253766601680	1.68764747416919	6.87206508750403

I4, singlet

Fe	1.17502151758657	4.38564895104736	2.82723312949155
P	2.92012670937459	5.69403672230971	2.57557823222533
P	0.10008910179463	2.47483863846622	2.95551569219298
P	2.18287421552902	3.71514103172198	4.63942493612491
C	3.68745685586654	5.73975235480391	0.93417366125226
C	2.83369553726622	7.45339022471236	3.02635227502360
C	3.92932666312176	4.14256045829121	4.55086836555042
C	1.03557866829194	1.38815915721563	4.06395583883098
C	1.70629909640731	4.31697923496319	6.28897086207215
C	2.00199751100108	1.93138566823521	4.80316424871905
C	-0.04408790933352	1.50710743555176	1.43024161658992
C	-1.58203094055618	2.40589936601057	3.64260104109223
C	4.24336724711940	5.06470435559631	3.64167446954507
N	-0.02325809482231	5.53625286659835	3.67045635601427
N	-0.72254101401853	6.21007879688183	4.17714339144992
H	2.12906369876458	3.47371459828294	2.14145390741655
H	1.83658582020059	5.39954133398649	6.34285346164208
H	2.31054193028952	3.85880264244050	7.07463210570773
H	0.65772815279598	4.08152999533551	6.48045699726839
H	-0.70062126670724	2.02238447820183	0.72564407226719
H	-0.45911802853488	0.51531412284224	1.62170106537940
H	0.93783127100891	1.40263754472271	0.96758221716350
H	3.00986326053362	6.21279951359812	0.21996606807255
H	3.88967310111546	4.72425235130698	0.59192419621240
H	4.62038742850826	6.30706474858372	0.94669984760342

H	2.12371114675286	7.96293283011662	2.37084136904189
H	3.80392492024799	7.94521052826685	2.92856414552721
H	2.48806748830698	7.56906235263275	4.05497645715658
H	-2.26594702486376	2.95409073617345	2.99055955401366
H	-1.60812470330063	2.87233308907141	4.62872663599173
H	-1.94348952350052	1.37932613694622	3.73349295367968
H	5.25735517567121	5.45138088745004	3.54128503548754
H	4.66138734284703	3.72998848123508	5.24312825587387
H	0.78812431094222	0.32884817419801	4.12910030967802
H	2.59171835950777	1.35295618455006	5.51233948681901
H	0.89983532375753	5.01928929437031	1.40056976962863
H	0.35074665102790	4.38909471328226	1.47801797219406

I5, singlet

Fe	1.45572173976949	4.02427751083769	3.40197846438086
P	3.26648914316636	5.27274136673594	3.15208283717408
P	0.29111007127129	2.16556677111283	3.69989779180494
P	2.04305100963463	3.82555827871666	5.46511876888175
C	4.31788219300644	4.91865521124830	1.71847506370416
C	3.19939826525941	7.09068995812128	3.16713644145073
C	3.79266363354185	4.22954679696791	5.60591778191730
C	0.96933084351640	1.39830210969163	5.19515848174581
C	1.28819747758130	4.78500381782615	6.81144845665736
C	1.77318250683824	2.11989174579269	5.97750355871941
C	0.45079855877500	0.88217309596047	2.42969808364138
C	-1.49706714567994	2.18611148141252	4.03335232973148
C	4.31899803197334	4.89877671662025	4.57925150405701
N	0.13723624023491	5.31804403541024	3.58038646853621
N	-0.64363924591233	6.08193863966574	3.67221469868402
H	2.49819262617381	2.98268576570216	3.15859776209108
H	1.43359358897199	5.85210566590181	6.63290851373256
H	1.72616349777559	4.53542379971725	7.78011826257204
H	0.21655588727234	4.58173554000015	6.85600132218650
H	-0.00243586928318	1.22557618783726	1.49725852827225
H	-0.04624737603164	-0.04257191177220	2.73040731614460
H	1.50543118761730	0.67886348003572	2.24131668356533
H	3.79102204302198	5.18199917300734	0.79872366941647
H	4.54972480919320	3.85366969276218	1.68539509164774
H	5.24851856222976	5.48890129894372	1.75354501989500
H	2.66550132658501	7.44424741968326	2.28199011670204
H	4.19616731967448	7.53673477590239	3.16651561680596
H	2.66169093371610	7.44276301539104	4.04903881412840
H	-2.03020648432059	2.53853709517124	3.14725677742557
H	-1.72129939589081	2.86751902786579	4.85568479917203
H	-1.87749675814672	1.19481114114651	4.28924294957835
H	5.35443876137600	5.23883356739378	4.59189281343473
H	4.36743804605459	3.99621521081765	6.50078299718521
H	0.69916196850271	0.37298496485201	5.44738327804448

H 2.19161200253163 1.72281755352049 6.90094893691286

I3, triplet

Fe	-0.42345236481736	-0.38607960235320	-1.04768883357160
P	-2.40729819315326	-1.10195384639844	-0.36244380293436
P	-0.41412400650332	1.83875707516544	-1.09016144160607
N	0.76306444774394	-2.78454339509419	-2.24530628560159
N	0.30567985948654	-1.89840518397227	-1.76347067280534
C	0.94989524218532	2.70143252309073	-1.96323102210310
C	-1.82526147650685	2.88748303588068	-1.60070526857556
C	-2.98131586574761	-2.78924722507688	-0.80153184925448
C	-3.98578198821371	-0.18755917755861	-0.59013394771716
C	0.11077984932807	1.52163850291917	1.56419364478439
C	-2.39142071897830	-1.22157719852659	1.45896438072794
C	-1.28774208713915	-0.83899566389989	2.10088256930529
C	1.52613850208396	-0.85745810851377	2.05277112412965
C	-0.13384163958899	2.42331629201854	0.61256659921037
P	0.10019463380886	-0.24134982183817	1.07354841084242
H	0.83295797236050	2.56663567105775	-3.04095421429413
H	1.90293623449007	2.25664964806974	-1.67192379800287
H	0.97944448608502	3.77259772751189	-1.74527964262676
H	-4.19048735560001	-0.07596483779897	-1.65736028166290
H	-3.89296055923805	0.81098078862641	-0.15925975866530
H	-4.83806318421757	-0.68884717814187	-0.12295465657795
H	-3.19180350098498	-2.83129735701521	-1.87299971450548
H	-3.88125825262980	-3.09093850444055	-0.25827299631918
H	-2.18584865413267	-3.50723349393842	-0.59300550378257
H	-3.26962100870422	-1.57812936271698	2.00010786706722
H	-1.21882335576514	-0.87287330644853	3.18892056816067
H	2.44946957365983	-0.44358088151899	1.64243117602896
H	1.57807969143675	-1.94417124155771	1.95829142597575
H	1.46712797823416	-0.60017073533003	3.11412554421112
H	-0.15595167444177	3.49204374434661	0.83275870225469
H	0.30119277368213	1.82765940725121	2.59373154452193
H	-2.02343850254420	2.73169614249366	-2.66327655062902
H	-1.63744007314233	3.95168502395589	-1.43312818409753
H	-2.72008678253574	2.59663053975150	-1.04923513188749

I2, singlet

Fe	-0.63762096387299	-0.30029269556039	-1.04879222827512
P	-2.48601992976251	-1.02622067708400	-0.32926803823030
P	-0.48079586799645	1.82797333875023	-1.05813914800850
C	0.82570541376094	2.64920632332573	-2.05429863157409
C	-1.89479792050011	2.91345978080247	-1.50168587663474
C	-2.99713975594402	-2.74167258611633	-0.73433757023605
C	-4.08865322571960	-0.17539065904429	-0.60944753416073
C	0.16100369480853	1.56999679050987	1.54350426706142
C	-2.48759363528379	-1.11319864289952	1.49483904113199

C	-1.37225411291857	-0.72632486094482	2.11305273649966
C	1.42516487563037	-0.97349997275696	1.72907245294091
C	-0.06371682211756	2.47537732208574	0.59281648383725
P	-0.03852346712348	-0.15373989841260	1.01342037577660
N	0.53914680630992	-1.61452566565065	-1.37783996915678
N	1.25545878803160	-2.43995790588882	-1.56272343129839
H	0.64192828751555	2.45434034695328	-3.11359817967570
H	1.79188158833608	2.20970885924660	-1.80197568921636
H	0.86882279395681	3.73161577721383	-1.90090928688134
H	-4.27091929011226	-0.09946110769679	-1.68379262660549
H	-4.03892362810296	0.83790407673472	-0.20800147916445
H	-4.93251990906794	-0.69626054120725	-0.14823950092042
H	-3.18579353826406	-2.81406895701707	-1.80814872777488
H	-3.89769277159210	-3.05751043991847	-0.19997263756708
H	-2.18173316641940	-3.42701669910682	-0.49642187678192
H	-3.36174217696356	-1.46163618999351	2.04653008251528
H	-1.26839542710461	-0.73862602306665	3.19809209427748
H	2.32325319551642	-0.61800772262450	1.22083726144129
H	1.35163748730038	-2.05112660071077	1.57113688125920
H	1.52470406955511	-0.78244456109138	2.80010425749901
H	0.00668135181783	3.54717043726459	0.78135100504193
H	0.42701609717286	1.83807176907099	2.56566443227287
H	-2.21749771894590	2.68091774092815	-2.51939756951569
H	-1.65152515050428	3.97842388158838	-1.45048345841681
H	-2.73313391966526	2.70757470542686	-0.83476037743002
H	-1.36551620444247	-0.49660895034958	-2.39850095803852
H	-0.58248584728848	-0.03244979276029	-2.56157657599149

I1, singlet

Fe	1.57687054438591	3.92448171954900	3.31915318312756
P	3.35397265782885	5.21262404051854	3.24450669014618
P	0.39702524475838	2.05795266140961	3.78736627595361
P	2.11005922215043	3.70652864554951	5.45487659536551
C	4.57383654481427	5.05218248029112	1.90563505349921
C	3.09502993441491	7.01726299692566	3.25348952754887
C	3.78707047375258	4.28644616125810	5.73545924094555
C	1.17547333312955	1.25704061043499	5.14035511860571
C	1.10737351673043	4.72619131918831	6.58685926520766
C	2.01016865910461	2.03329516217996	5.93397247858450
C	0.10291849803330	0.74501226729867	2.56236935180961
C	-1.32529961448300	2.45316788291200	4.24287419192337
C	4.35505760153573	4.95830575016355	4.72463839329364
N	0.26989768109985	5.13440526704355	3.44428643505385
N	-0.52767022533652	5.88942594897395	3.55211041226027
H	1.59302765134799	4.08643549889268	1.70500262423100
H	1.44345541976671	3.26623878909081	1.83885475268905
H	1.13353500207340	5.77305578528819	6.27465379361826
H	1.46250363100476	4.64872106619987	7.61591267638687

H	0.07376899820263	4.37601454477504	6.55236610943207
H	-0.50188893797003	1.12878144418584	1.73812816072477
H	-0.42255364591010	-0.09791938498274	3.01817521055286
H	1.05718738309327	0.39715427140534	2.16424546358890
H	4.10673018538147	5.32279002383881	0.95623100975070
H	4.90203310096912	4.01376988980073	1.84196034288128
H	5.44110217948745	5.69700114808538	2.06871338015207
H	2.57956890986707	7.31126497669866	2.33606380234485
H	4.03448224978531	7.56983982222130	3.32779587468147
H	2.45849140062774	7.28308816185692	4.09919173660002
H	-1.83862207334971	2.92269932808899	3.39946980770552
H	-1.32765155823136	3.15721194787263	5.07618333406424
H	-1.87190478865236	1.55675063988991	4.54294479449421
H	5.38339548477920	5.31498729979975	4.77113142898104
H	4.32108576781586	4.07503351674135	6.66019853903759
H	1.11091749432563	0.17911156124715	5.26047758084675
H	2.65682207366620	1.60226675530578	6.69292736391015

MECP

Fe	-0.57458105676818	-0.34499010706556	-1.14211239698030
P	-2.48504673363630	-1.06098707401665	-0.36054243892264
P	-0.45812765696835	1.90192228706152	-1.09626840360444
C	0.84211503060052	2.77907286793324	-2.05069498824137
C	-1.89783815926322	2.96655030494812	-1.48745948631167
C	-3.03094888490964	-2.77765426597526	-0.72017353270012
C	-4.08720199598419	-0.19299079846081	-0.60387858527912
C	0.18507269332964	1.55214531703229	1.52303141012118
C	-2.44772181414531	-1.12114566332237	1.46277439723741
C	-1.33090546063506	-0.74039744719109	2.08282839772293
C	1.47577105914654	-0.92171309416704	1.93177912596735
C	-0.05304300742968	2.46934556284186	0.58470124736522
P	0.05305623373504	-0.19601002490500	1.03139979053614
N	0.44378094186698	-1.86521082645568	-1.51945325834551
N	1.06305065468597	-2.76495423726926	-1.69952675625115
H	0.65088752661063	2.65329517807781	-3.11902413770429
H	1.81207146535289	2.32908012875057	-1.83243063831499
H	0.88828207053657	3.84900221221769	-1.82740526661372
H	-4.30390199274775	-0.13310964065454	-1.67300443552510
H	-4.01167841870339	0.82641924000976	-0.22177853911924
H	-4.92369012727355	-0.69228806304330	-0.10662151454224
H	-3.25374963584860	-2.86705392119506	-1.78625282501303
H	-3.91752239660432	-3.07701311208895	-0.15342518412025
H	-2.21642962173216	-3.46869493167248	-0.49499987433834
H	-3.32166595500215	-1.45499766282703	2.02470235887984
H	-1.24639376532631	-0.75123578002399	3.17028935234913
H	2.40475549114525	-0.55203630753064	1.49316453255937
H	1.45899723164941	-2.00663625733101	1.80816086485294
H	1.47527801991662	-0.69164500624810	3.00086915114817

H	0.00304047880922	3.53614654026142	0.80769462438679
H	0.44468324739256	1.83730895856416	2.54329857149530
H	-2.20838275061514	2.78248920517881	-2.51855127436573
H	-1.68342855349368	4.03289529341042	-1.37322978932683
H	-2.73331768934176	2.70191906122487	-0.83809975608990
H	-1.37377570101079	-0.55947446263425	-2.68608495135033
H	-0.73408076733819	-0.09565347343450	-2.88556579156142

TSE, singlet, Freq= -237.74

Fe	1.20262426181713	4.28436495783112	2.22772789426644
P	2.44378888797201	6.02429873779945	2.52215166814797
P	0.68615017161131	2.21162054731832	1.91992754852348
P	1.91303017805058	3.64615610293699	4.15692852736390
C	3.58021296575239	6.59195000060759	1.21705738576548
C	1.73567547258390	7.61578467214845	3.07265152106930
C	3.42033644275469	4.54704130844541	4.58007093032970
C	1.60426473130055	1.21566860399187	3.14091557182134
C	0.94453147111793	3.82559098830151	5.69494973740331
C	2.21408524826552	1.86508726072368	4.13412760325876
C	1.12612821281366	1.36393811616769	0.36852877216327
C	-1.02095145035933	1.61030856198542	2.16396809584862
C	3.61867829292182	5.65767055423773	3.86791553508607
N	-0.39959409869358	4.94887582560389	2.74173740320607
N	-1.37143741147997	5.34821600179053	3.07143325996654
H	0.89718567110747	4.65199407479744	0.75897503647470
H	2.52763394957000	3.75451991771662	1.72709874857360
H	0.71905901578414	4.88004511035860	5.86569119139228
H	1.47923383876714	3.44143113564189	6.56635279574620
H	0.00201587161710	3.28258474259263	5.60139584317529
H	0.54634408963513	1.78828898206245	-0.45299365086519
H	0.91753540586220	0.29263301773792	0.41938390381923
H	2.18931515985615	1.50105329671463	0.16631048131387
H	3.00047646595248	6.94668689694708	0.36317007102179
H	4.21029449511019	5.76124074538548	0.89741707436111
H	4.21897752929132	7.40767713677456	1.56309454322857
H	1.09651023007481	8.01837439228695	2.28320179071105
H	2.50213675551520	8.35676925036313	3.31051228557791
H	1.11398121359228	7.46198968281869	3.95638437934764
H	-1.66900806522276	2.06516152365991	1.41163833723610
H	-1.38830547085275	1.91235516052956	3.14642496181667
H	-1.10047812742027	0.52366399631289	2.08313621961123
H	4.44688848969114	6.33357310561898	4.08225001737999
H	4.06601872639020	4.26123647268924	5.40894247886833
H	1.63708977805651	0.12846564968900	3.06497730389481
H	2.76710496862728	1.34851494108514	4.91705748245633
H	0.88184345692408	7.56580688886508	-2.86335494345537
O	1.42310544578650	4.39286396849028	-1.80648774268006
C	2.79966627121717	4.11676925641892	-1.38622107989698

C	3.40651431784321	3.04985959071033	-2.25527246967835
H	3.35366065217541	5.05821732105185	-1.43308380595492
H	2.71362739178769	3.83768402596628	-0.33010354075846
H	4.42497439775308	2.86206459440712	-1.90893290070229
H	2.85708544118208	2.10930253382293	-2.19011569019747
H	3.47441509467381	3.34294124426603	-3.30533903677648
C	1.14520770245562	5.46111969908672	-2.75442858561871
C	1.18592425196601	6.82679189825817	-2.11950289784510
H	1.87979828087258	5.34636374857424	-3.55539333029145
H	0.16226696125578	5.23166753090079	-3.16769908724283
H	0.49871959790868	6.90003604328938	-1.27421411177676
H	2.18496916107965	7.10465220508377	-1.77937480220792
H	-3.59951033000207	2.60212095131719	-1.20481682272037
O	-1.12959828064997	3.98799356904968	-1.20574753142658
C	-1.98987339385526	4.83744476539280	-0.46963495466441
C	-2.76049832913134	5.83422378189628	-1.31383519336565
H	-2.68381641910256	4.24742193446233	0.14535208121795
H	-1.33314457528217	5.35950858421331	0.22985170901407
H	-2.09771395794543	6.44692979768973	-1.93140437520988
H	-3.49807251435902	5.36655551356935	-1.97010230076335
H	-3.30871461852835	6.51130949260925	-0.65576209438990
C	-1.71271502241901	3.13629004068934	-2.17148434870436
C	-2.70366278395184	2.13224436803358	-1.61635988069356
H	-2.18896388412047	3.72332163768514	-2.97179861383034
H	-0.87837678511514	2.60187053018843	-2.63954044592435
H	-3.03392937743768	1.47130174101262	-2.42019494808206
H	-2.25700572162992	1.50675810092428	-0.84001066544059
H	0.73025420121409	4.31111317039564	-1.08949033929608

TSF, singlet, Freq= -2893.58

Fe	1.64146211904880	4.16374466095001	2.73132703564723
P	3.40801494849668	5.49971274720644	2.74622866162841
P	0.54564087061805	2.24355527745054	2.85202110515944
P	2.08671291821600	3.84080993951092	4.82693316770315
C	4.60740157010971	5.31806307003919	1.39762123596460
C	3.22583037924065	7.30556535981062	2.88007110524032
C	3.79386901453118	4.31200082018150	5.15416994144977
C	1.13408414613699	1.40167299055486	4.34568842447204
C	1.17673127395130	4.68158523702183	6.15939961562880
C	1.85323737685920	2.09993043751857	5.22447496972203
C	0.83482191819528	1.03034597122943	1.53447033640959
C	-1.26257042435979	2.18698858715544	3.05224292824898
C	4.36441412848587	5.08365816579368	4.22872362806222
N	0.25513220468106	5.39617364605384	2.96558762042261
N	-0.55589470699223	6.11750282663595	3.11616213682310
H	2.74999944141873	3.17349646357256	2.50806336838066
H	1.28904091802743	5.76300708074111	6.06119987531675
H	1.54154343133093	4.38619543445626	7.14531088989320

H	0.11534340220571	4.43404750717667	6.09743086252510
H	0.40195181104552	1.38768968592632	0.59745120736087
H	0.37916422038203	0.06692962237819	1.77301271873053
H	1.90633658834748	0.89282971681713	1.38566163831244
H	4.16696872614443	5.66118237702021	0.45872334264325
H	4.87743466695910	4.26761102168644	1.28401791112125
H	5.51030770052563	5.90351220272951	1.58365311304344
H	2.74065859442162	7.69057413305924	1.98017582447715
H	4.18878325255932	7.80806413572445	2.99364458793583
H	2.59858768205256	7.56077250842926	3.73590621649567
H	-1.74343831274528	2.57972832971503	2.15333980551855
H	-1.57173086913000	2.80376017643674	3.89780562280162
H	-1.62308632907813	1.16893088348752	3.21537471528522
H	5.37210345127801	5.47902891631589	4.35572572637429
H	4.29908356970302	4.04115204863356	6.07987739292830
H	0.87835461168219	0.35617174167050	4.51679933926001
H	2.21743760396207	1.66651563404467	6.15452550545231
H	1.19110208392566	3.97912636375902	0.79456432474101
H	1.72324601776308	4.54937427910659	0.77094409882000

TSG, singlet, Freq= -151.23

Fe	1.48922357288902	4.03508305823170	3.40679002234726
P	3.32020676812339	5.26755697308855	3.17981463515518
P	0.31260969061192	2.18457200628879	3.71129033570426
P	2.09044603108932	3.80196318750025	5.46689900077808
C	4.41171105400349	4.92115084130863	1.77252851196919
C	3.25915016022134	7.08636868380817	3.19764741284062
C	3.83795430441948	4.19962866903882	5.62514453811710
C	0.97820474869405	1.39725651882659	5.20169806920597
C	1.33315383543033	4.76211325776079	6.81279385506956
C	1.80299269075845	2.10027775437311	5.97838658860676
C	0.44527911255990	0.88896503070547	2.45102176527917
C	-1.47509659184892	2.23751664600095	4.04621441412192
C	4.36446826355172	4.88741050821231	4.61177466467771
N	0.19738690585745	5.34170492547040	3.68742779401891
N	-0.56419290626801	6.11148329430400	3.85839476537785
N	0.70552707246473	3.93881739312539	1.00987810095505
H	2.51635412229699	2.98497835408798	3.12476623824652
N	1.20174819106677	4.83276327694852	0.62927833970601
H	2.73410992619715	7.44074492902026	2.30756340040982
H	4.25750545211422	7.52871195367194	3.20619819447608
H	2.71456762161269	7.43992376034811	4.07473101052473
H	3.94048775806364	5.24014675876467	0.84107435552064
H	4.60612223699505	3.84985346201643	1.71134598662390
H	5.36097989989509	5.45248282802714	1.86925983593114
H	-0.03736384954683	1.21612345855675	1.52860609183915
H	-0.03690918049905	-0.03364894674285	2.78086995126673
H	1.49560856364060	0.68905739328131	2.23683505255369

H	-1.99997181058644	2.59985883419119	3.15912995041171
H	-1.68698725493838	2.92334811250104	4.86810460846465
H	-1.87519189164893	1.25372413473256	4.30106638214924
H	0.25974505951863	4.56704552214026	6.85158003456834
H	1.48929856412335	5.82916799997209	6.64322999680655
H	1.76394773916405	4.50204227173262	7.78195560562389
H	5.39766357864657	5.23378653033364	4.63277505722435
H	4.40691299314158	3.95357451308493	6.52024463381300
H	0.68869987130007	0.37687303077009	5.45213034527312
H	2.22334769688483	1.69630307451592	6.89791045434050

TSD, singlet, Freq= -170.65

Fe	-1.51149321291383	0.28976557450676	-0.72481983463764
P	-3.04175885168685	-0.96316416121475	0.14485932932382
P	-0.43272525768742	2.15807812791304	-0.86704952639667
N	-3.07819313328406	0.91687927730692	-3.08547133218889
N	-2.51062348563247	0.69741292678023	-2.16141489432725
C	1.15040773006204	2.14747693958622	-1.78899123014793
C	-1.16123947004238	3.68610961885952	-1.55435337664890
C	-2.80150314885366	-2.77656735347009	-0.00658123518005
C	-4.82074728831866	-0.90469568783267	-0.28127304369414
C	0.03032123991559	1.81847876496210	1.76578624319986
C	-3.02488532153883	-0.82300971027116	1.96427098241333
C	-1.93791893892471	-0.25852139444684	2.49323205544744
C	0.67593419532881	-0.89383852633029	1.72598117851605
C	0.16985533108704	2.69372605868418	0.76879628572836
P	-0.69314162915821	0.23019695052769	1.26359160988028
N	-0.40123317887089	-0.89604013216869	-1.51921126418418
N	0.28019935061393	-1.61892090375246	-1.99660386598807
H	0.95012482023784	1.92501818356173	-2.83998109600385
H	1.79398832401724	1.35593176941325	-1.40130288924788
H	1.68808979278562	3.09719143719841	-1.73177474274880
H	-4.93379509984420	-1.08576847452580	-1.35259619681491
H	-5.25625967263848	0.06828342557143	-0.06054658315458
H	-5.39042182954295	-1.66947836089128	0.25205395811619
H	-2.89720177457427	-3.06597260427278	-1.05613329558087
H	-3.52138601002603	-3.35218358961246	0.58031805020988
H	-1.79430646903872	-3.04297736558469	0.31769786472465
H	-3.80643344135391	-1.26960353088912	2.58004074180210
H	-1.75824513109438	-0.21423438141934	3.56694277672143
H	1.54409815951261	-0.67895958303279	1.09974393638410
H	0.37576263992656	-1.92800120237859	1.54666112382186
H	0.96843067866811	-0.79400086339638	2.77360585740889
H	0.67870201809930	3.64921435091756	0.89980805708913
H	0.41970293781992	1.99709907336503	2.76757783832937
H	-1.42251584142219	3.51869154403400	-2.60133400183223
H	-0.46935852244581	4.53051973577478	-1.50831840260277
H	-2.07784513031686	3.94244560186746	-1.02414109898056

H	-4.25414395323969	5.63967017291457	-3.51070418271184
O	-5.08473795334673	2.93543520957827	-1.66196479125568
C	-5.96264555890121	2.17511134067488	-2.47001129367921
C	-7.41362791051903	2.61634663559354	-2.40845696239598
H	-5.61479631287894	2.15942682465530	-3.51096716520180
H	-5.87505549162867	1.14333706884979	-2.11728572335968
H	-8.02718262472203	1.91707757026631	-2.98011132345020
H	-7.80124453678222	2.62788696066141	-1.38600556918302
H	-7.58013326493515	3.60569925582637	-2.84095252976030
C	-4.95070968654635	4.30122455146023	-1.99057372272856
C	-4.47786189195067	4.57623147813486	-3.40451107972522
H	-5.89045134708275	4.84346082079613	-1.80139820968827
H	-4.22063681673899	4.70826115918726	-1.28179735487069
H	-3.57098836897651	4.01628484130800	-3.64218045877507
H	-5.22973116413755	4.33561742812754	-4.15905922487363
H	-5.73493278555511	3.01628816875862	3.40247225770621
O	-4.36094839641623	2.31851113567899	1.05434950300945
C	-3.30780854024951	2.90432608833066	1.88367438802691
C	-3.29809791802446	4.40854561423787	1.86100683302878
H	-3.44525538123524	2.49045440251100	2.88388613643896
H	-2.40198198255577	2.47009594175941	1.45766155021665
H	-3.17573754120310	4.80963212115490	0.85275748643246
H	-4.18306810258352	4.85969208436524	2.31453254581332
H	-2.43748394110129	4.74292916872880	2.44409478139110
C	-5.76847465897669	2.66205891576649	1.25026943455831
C	-6.19825533180424	2.36332097086070	2.66075789986702
H	-5.91150596818044	3.71049320611434	0.97518063636829
H	-6.30758002198817	2.04776208544271	0.52975616064003
H	-7.27531327894643	2.52991726153292	2.72523209495552
H	-6.01097436645394	1.32344418031541	2.93537662336335
H	-4.07718241869569	2.14951295976263	0.12294379568664

TSC, triplet, Freq= -108,97

Fe	12.29542457234069	5.09808052541515	11.40774322345259
P	10.33780654962353	5.04483359188491	10.19631756968750
P	12.51898381763786	2.96355406772865	10.66086176567010
P	12.72485564894584	6.94144096945079	10.15627158995189
N	11.58769073834550	5.37474367568921	13.12448117911844
C	10.39953128039871	6.30174758817539	8.87672799843478
C	8.58012266438604	5.20726145639928	10.70581558320867
N	11.24841223555168	5.53884285208914	14.17575149252875
C	11.43499916280778	7.14287648510167	8.88584404271105
C	10.24532742639525	3.49224127960930	9.24526314142555
N	14.36382163115583	5.11958181792304	12.44475584762099
C	11.18888047908605	2.57828037997125	9.47762565123641
C	12.42646623449918	1.51785010775255	11.77837167302777
C	14.23115737317414	6.99631864755712	9.11126182602021

N	15.45860978544596	5.06100751598384	12.46279161343308
C	12.76583980120334	8.61934219272887	10.88480564475726
C	13.99242551054099	2.52212308946928	9.66214940352753
H	7.87302849946810	5.13459138767091	9.87468229688140
H	8.44328161075235	6.17062903439991	11.20114725025369
H	8.35046533023502	4.42545284327753	11.43261388479526
H	12.85279675084377	9.40800676149716	10.13179956528575
H	13.61366232056305	8.69173723165144	11.56952938911910
H	11.85642932506105	8.77777299604161	11.46637368611047
H	12.42179542645072	0.56531798029493	11.24068353904195
H	11.52147353916297	1.59162738567000	12.38328361240053
H	13.28148923120924	1.53563925686640	12.45748172497651
H	14.10461973895739	3.23575851906519	8.84398804461221
H	13.93353271397535	1.51411498852138	9.24269836025707
H	14.88693667763958	2.58410390061639	10.28644429231595
H	14.26321092803980	7.87749705294442	8.46481241421615
H	14.27678379046296	6.10202633379945	8.48692600056037
H	15.11769848048335	7.00475325526119	9.74970957326578
H	9.43702429235786	3.29744298939816	8.53881170051575
H	11.17855434407975	1.61354564090806	8.96699356918719
H	9.61046313403286	6.39160540810479	8.12862705706300
H	11.52296895468233	7.94116079107997	8.14662479332579

TSB, triplet, Freq= -272.65

Fe	-0.40217184411270	-0.35342146888778	-1.01199155250195
P	-2.48987627375685	-1.01359593637422	-0.30029304579545
P	-0.57565428302546	1.92575189748107	-0.99002550159894
C	0.82538159654947	2.70148462327214	-1.89998176748341
C	-1.96499622926410	2.82447289759079	-1.75968811688736
C	-3.19226847447979	-2.61646083729450	-0.84098396204848
C	-3.97796854736932	0.04326476149084	-0.39488918057322
C	0.11251502303055	1.55773753098566	1.59649000205532
C	-2.29204993287150	-1.23504544587916	1.47012527092515
C	-1.11125128123118	-0.94883840002074	2.05775715322894
C	1.70871471591474	-0.81550342813356	1.88999415825740
C	-0.49510609372504	2.43570291200774	0.68208423618488
P	0.20442023513206	-0.12659133189697	1.13960293605294
N	0.26372456453674	-2.03787177248511	-1.16645079451199
N	0.68855232980253	-3.05052782026538	-1.27438028624469
H	0.80129245539574	2.44818112894451	-2.96423415133862
H	1.76315092327793	2.33565957217791	-1.47799427828819
H	0.80700481651359	3.78984014475808	-1.79703108582196
H	-4.25961991415285	0.21758497387633	-1.43606332409613
H	-3.75780328365587	1.00398278372416	0.07360344797060
H	-4.82676419787662	-0.40757583718104	0.12614319168424
H	-3.49560890806569	-2.55760094083676	-1.88932242028163
H	-4.06262429035247	-2.90641277007131	-0.24603199333866

H	-2.43775615847303	-3.39993963827351	-0.75111444351395
H	-3.14465220592556	-1.57231489832323	2.06108885524302
H	-0.99811339164068	-1.07016860351595	3.13633113181826
H	2.58641333021887	-0.35255600362852	1.43576850024963
H	1.75530842411893	-1.89529901725502	1.73069135046225
H	1.73651566407218	-0.62101434016019	2.96526970764374
H	-0.74935074296271	3.45549286474941	0.96386481724652
H	0.30582722801752	1.86294666275502	2.62304026744050
H	-2.00090368634864	2.62507890995787	-2.83325872550295
H	-1.85315744239498	3.90247243420962	-1.61552402151540
H	-2.91281765639240	2.51684540754964	-1.32021871561108
H	-1.16814246724354	-0.52199810623610	-3.25907971459240
H	-0.48275400125854	-0.26206290881203	-3.45518794491665

TSA, singlet, Freq= -242.35

Fe	0.25772471881319	-0.39155896296561	-0.79785580278088
P	-1.48381135545310	-1.36478331808613	-0.02149010417410
P	0.70383521602517	1.68765267285182	-0.82627672559362
P	0.87943259449949	-0.31428683391200	1.25536044518670
C	-3.16364517969125	-0.74375656886682	-0.41788612822013
C	-1.77536870502936	-3.16074677371624	-0.29849699125152
C	-0.42213224500147	-0.85859824277978	2.39469860443123
C	1.18975514021512	2.29121123166308	0.82324524721532
C	2.32919854282189	-1.22573840730050	1.89907458783718
C	1.26447095603652	1.37747540658317	1.78913830488010
C	-0.55254576839350	2.94368523087247	-1.28391941985880
C	2.11299315207537	2.31899585686809	-1.82334354616283
C	-1.51222676304478	-1.33971767349555	1.79960079184501
N	1.64479004786335	-1.35899474213619	-1.37321175921049
N	2.51670537712246	-1.94735782270898	-1.72071622073014
H	-0.32167158878418	-0.64885130515603	-2.22532249219255
H	-0.65240140553885	0.13035575215299	-1.91279687438923
H	2.18654660895062	-2.29500334062488	1.73147932483069
H	2.48952302254245	-1.05461300780321	2.96635341482051
H	3.22362767661632	-0.91462540286690	1.35564001079245
H	-0.85443991048277	2.78737958167027	-2.32252520025501
H	-0.18496329054732	3.96881437842848	-1.18332355952798
H	-1.43665554246561	2.81626218965636	-0.65715883260067
H	-3.31640094985009	-0.79940712628946	-1.49856251330295
H	-3.24093441246513	0.30444209423418	-0.12499117823441
H	-3.95782930515774	-1.31492081642055	0.07107515364040
H	-1.91140280188261	-3.33571445736766	-1.36842855233437
H	-2.65040994171713	-3.54606983100833	0.23316855694058
H	-0.89144182309103	-3.71813156550885	0.01628919216780
H	1.91163475047334	2.13003421668546	-2.88059943554407
H	3.01691936512325	1.76782591885918	-1.55772934518341
H	2.29392721002835	3.38937076279124	-1.68890730913648

H	-2.36281949849142	-1.72465156432600	2.36324862853006
H	-0.30545828825168	-0.81834051496653	3.47736629340763
H	1.41581759918593	3.34379794343907	0.99778345155186
H	1.55965679694621	1.61856504155028	2.81001998260612
